# Antagonism of Protease Activated Receptor-2 by GB88 Reduces Inflammation Triggered by Protease Allergen Tyr-p3

**DOI:** 10.3389/fimmu.2021.557433

**Published:** 2021-09-08

**Authors:** Yun-Ju Wang, Sheng-Jie Yu, Jaw-Ji Tsai, Ching-Hsiang Yu, En-Chih Liao

**Affiliations:** ^1^Institute of Biomedical Sciences, Mackay Medical College, New Taipei City, Taiwan; ^2^Department of Medical Education and Research, Kaohsiung Veterans General Hospital, Kaohsiung, Taiwan; ^3^Division of Allergy, Immunology & Rheumatology, Department of Internal Medicine, Asia University Hospital, Taichung, Taiwan; ^4^Division of Allergy, Immunology & Rheumatology, Department of Internal Medicine, Taichung Veterans General Hospital, Taichung, Taiwan; ^5^Department of Medicine, Mackay Medical College, New Taipei City, Taiwan

**Keywords:** storage mite allergy, *Tyrophagus putrescentiae* (schrank), protease activated receptor-2 (PAR-2), GB88 (PubChem CID: 73755230), protease allergen

## Abstract

The occurrence of allergic diseases induced by aeroallergens has increased in the past decades. Among inhalant allergens, mites remain the important causal agent of allergic diseases. Storage mites- *Tyrophagus putrescentiae* are found in stored products or domestic environments. Major allergen Tyr-p3 plays a significant role in triggering IgE-mediated hypersensitivity. However, its effects on pulmonary inflammation, internalization, and activation in human epithelium remain elusive. Protease-activated receptors (PARs) are activated upon cleavage by proteases. A549 cells were used as an epithelial model to examine the PAR activation by Tyr-p3 and therapeutic potential of PAR-2 antagonist (GB88) in allergic responses. Enzymatic properties and allergen localization of Tyr-p3 were performed. The release of inflammatory mediators, phosphorylation of mitogen-activated protein kinase (MAPK), and cell junction disruptions were evaluated after Tyr-p3 challenge. Enzymatic properties determined by substrate digestion and protease inhibitors indicated that Tyr-p3 processes a trypsin-like serine protease activity. The *PAR-2* mRNA levels were significantly increased by nTyr-p3 but inhibited by protease inhibitors or GB88. Protease allergen of nTyr-p3 significantly increased the levels of pro-inflammatory cytokines (IL-6 and TNF-α), chemokine (IL-8), and IL-1β in epithelial cells. nTyr-p3 markedly increased phosphorylation of extracellular signal-regulated kinase (ERK)1/2 and MAP kinase. When cells were pretreated with GB88 then added nTyr-p3, the phosphorylated ERK1/2 did not inhibit by GB88. GB88 increased ERK1/2 phosphorylation in human epithelium cells. GB88 is able to block PAR-2-mediated calcium signaling which inhibits the nTyr-p3-induced Ca^2+^ release. Among the pharmacologic inhibitors, the most effective inhibitor of the nTyr-p3 in the induction of IL-8 or IL-1β levels was GB88 followed by SBTI, MAPK/ERK, ERK, and p38 inhibitors. Levels of inflammatory mediators, including GM-CSF, VEGF, COX-2, TSLP, and IL-33 were reduced by treatment of GB88 or SBTI. Further, GB88 treatment down-regulated the nTyr-p3-induced *PAR-2* expression in allergic patients with asthma or rhinitis. Tight junction and adherens junction were disrupted in epithelial cells by nTyr-p3 exposure; however, this effect was avoided by GB88. Immunostaining with frozen sections of the mite body showed the presence of Tyr-p3 throughout the intestinal digestive system, especially in the hindgut around the excretion site. In conclusion, our findings suggest that Tyr-p3 from domestic mites leads to disruption of the airway epithelial barrier after inhalation. Proteolytic activity of Tyr-p3 causes the PAR-2 mRNA expression, thus leading to the release of numerous inflammatory mediators. Antagonism of PAR2 activity suggests GB88 as the therapeutic potential for anti-inflammation medicine, especially in allergy development triggered by protease allergens.

## Introduction

The occurrence of allergic diseases, such as allergic asthma, allergic rhinitis, and atopic dermatitis has increased in both developed and developing countries in the past decades (1). Allergic asthma or bronchial asthma is an immune-derived inflammatory disease of the airways that may evolve in susceptible individuals in response to aeroallergen exposure (2). Among the indoor inhalant allergens, domestic mites are the most common and important triggers of allergic diseases in tropical, subtropical, and humid areas (3). Taiwan is an island located in a subtropical area with optimal humidity and temperature for the growth of mites (4). According to the universal distribution and allergenic importance, domestic mites are mainly classified into two categories: house dust mites and storage mites (5). *Tyrophagus putrescentiae* is one of the widespread species of storage mites and has been found infesting various stored foodstuffs or stored products with high lipid and protein contents (6, 7). *T. putrescentiae* is not only found in stored products but also prevalent in the dust from domestic environments (8). *T. putrescentiae* might cause allergic respiratory diseases in urban areas and can cause allergic symptoms after occupational exposure in rural areas (9, 10). Approximately 38% of clinic patients suffering from allergic symptoms had sera containing specific IgE against *T. putrescentiae* crude extracts (11). It has been reported that *T. putrescentiae* caused systemic anaphylaxis after the consumption of foods contaminated with storage mites (12).

Mite allergen exposure is primarily the result of inhalation of fecal particles containing partially digested food and digestive enzymes (13). The allergens from the mite feces are mostly the major allergens and contaminants of the indoor environment because these allergens accumulate and persist in the domestic environment (14). The Der f 3 allergen with a trypsin-like protease activity is purified from the fecal extract of house dust mite *Dermatophagoides farinae*, which is involved in the digestive process of mite (15). Six allergens of *D. farinae* including Der f 3 were increased in the spent growth medium extract with a high amount of mite feces detected by two-dimensional gel electrophoresis-mass spectrometry (2-DE-MS) proteomic approach (16). The physiological characteristics and pH of the gut from storage mites are very similar to other species of house dust mites, indicating that *T. putrescentiae* possesses the analogous digestive enzymes and fecal properties as others (17). The extracts of *T. putrescentiae* feces contained higher levels of proteases such as trypsin or chymotrypsin compared to that in body extracts, and these proteases are involved in the digestive process in mites (18).

The protease-activated receptors (PARs) are a subfamily of G-protein-coupled receptors with seven transmembrane-spanning domains and designed as PAR-1, 2, 3, and 4 that are activated upon cleavage by proteases (19). Among these four receptors, PAR-2 is mainly activated by trypsin or trypsin-like serine protease through cleavage of the extracellular N-terminal domain to activate G-protein-coupled signal transduction pathways (19). Accumulating evidence suggests that activation of PAR-2 is correlated to the release of proinflammatory cytokines and the expression of adhesion molecules in inflammatory cells (20). The expression of PAR-2 is up-regulated in the respiratory epithelium of asthma patients (21). PAR-2-mediated activation of airway epithelial cells induces the release of GM-CSF (granulocyte-macrophage colony-stimulating) and eotaxin, which promotes the eosinophil survival and activation and mediates eosinophil recruitment to the airway (22). The PAR-2 activation promotes the proliferation of endothelial cells, the production of vascular endothelial growth factor (VEGF), and the expression of cyclooxygenase-2 (COX-2) in human endothelial cells (23, 24).

Although at least 37 groups of allergenic components have been discovered in the house dust mite, *D. pteronyssinus*, relatively few allergens of storage mite, *T. putrescentiae*, have been identified in the allergen database (www.allergen.org). Among these allergenic components, mite allergens can be generally divided into two categories as protease and non-protease molecules (25). Many protease allergens in house dust mites have been identified, such as groups 1, 3, 6, and 9. Among them, group 3 allergen exhibits serine protease activity (25). Der p 3 can induce the release of proinflammatory cytokines from human pulmonary epithelial cells through the interaction with PAR-2 (26). Furthermore, the mite allergens from house dust mites can cause the release of inflammatory cytokines such as GM-CSF and IL-6 from human pulmonary epithelial cells, and these cytokines then participate in the ongoing inflammation and structural alterations of asthma (27).

GB88 is an antagonist for human PAR-2 by inhibiting the PAR2-activated Ca^2+^ release which is induced by native trypsin or a synthetic peptide and non-peptide agonists (28). GB88 is also an orally bioactive anti-inflammatory compound and inhibits the acute rat paw edema elicited by proteolytic or peptide agonists of PAR-2 but not by PAR-1 or PAR-4 (28). An orally active PAR-2 antagonist GB88 is effective in treating chronic arthritis induced by collagen in rats through inhibition of macrophage infiltration, mast cell degranulation, and β-tryptase-PAR2 signaling in joint inflammation (29). The PAR2 modulators derived from GB88 have great potential for treating inflammatory and respiratory disorders through suppression of inflammatory cytokines secretion such as TNF-α and IL-6, induced by PAR2 agonists, in human kidney tubule epithelial cells (30). The antagonistic effect of GB88 derivatives has been demonstrated in inhibiting Ca^2+^ release and cAMP stimulation, suggesting that antagonists of PAR2 ligand offer anti-inflammatory properties both *in vitro* and *in vivo* (30).

The recombinant Tyr-p3 (rTyr-p3), which is one of the major allergens in *T. putrescentiae*, has been cloned and identified as trypsin-like serine protease in our previous study (11). The allergen of rTyr-p3 may play a significant role in triggering IgE-mediated hypersensitive reactions that can induce an allergic inflammatory response in the airways of susceptible individuals (11). So far, the direct evidence of internalization and activation of human epithelium by native Tyr-p3 (nTyr-p3) with trypsin-like serine protease activity has not been investigated. Therefore, we used the nTyr-p3 instead of rTyr-p3 to realize the association of human epithelium in this study.

The intercellular tight junction and adherens junction are the primary components of the epithelial paracellular permeability barrier, which offers a potential barrier for allergens to penetrate and interact with dendritic antigen-presenting cells. The ZO-1 is one of the families of tight junction-associated proteins that function as cross-linkers by anchoring the tight junction strand proteins to the actin-based cytoskeleton (31). Adherens junctions are constructed on a foundation of homophilic contacts between epithelial cadherin (E-cadherin) clusters on the surface of adjacent epithelial cells (32).

In this study, the integrity of tight junction and adherens junction be disrupted by mite allergen of nTyr-p3 in the airway epithelium was investigated. We aimed to examine whether nTyr-p3 protease from storage mite allergen can stimulate PAR (PAR-1 to 4) through its proteolytic activity in the human epithelial cell. We also determined the effects of nTyr-p3 on the production of pro-inflammatory cytokines (IL-6, TNF-α, and IL-1β), and chemokine (IL-8). We investigated the mechanisms of nTyr-p3 in the inflammatory process by studying the phosphorylation of ERK1/2 and MAPK as well as the release of inflammatory mediators. Finally, we examined the potential inhibitory effects of pharmacologic inhibitors including GB88 (PAR-2 antagonist), SBTI (Soybean trypsin inhibitor), phosphorylation inhibitor (p38 inhibitor, ERK inhibitor, and MAPK/ERK inhibitor) on the production of inflammatory mediators (GM-CSF, VEGF, COX-2, TSLP, and IL-33). These findings suggest that targeting PAR-2 by antagonists or Tyr-p3 inhibitors might prove to be beneficial in treating the protease allergen-induced inflammatory diseases.

## Materials and Methods

### Purification of nTyr-p3

nTyr-p3 was extracted using frozen spent growth medium (SGM) and purified with an immune-affinity column bound with an anti-Tyr-p3 antibody described in our previous study (11). Briefly, 10 g SGM were homogenized in phosphate-buffered saline (PBS, pH 7.2) by sonication and centrifuge. The supernatant was filtered with a 0.45 µm filter and loaded on an antibody-binding affinity column. Ten milligrams of rabbit anti-Tyr-p3 serum were bound to the swollen cyanogen bromide-activated-Sepharose^®^ 4B (Sigma-Aldrich, St. Louis, Mo., USA) by incubation in coupling buffer (100 mM NaHCO_3_ and 0.5M NaCl, pH 8.3) overnight at 4°C. The resins were washed with three cycles of solution A containing 0.1 M CH_2_NaO_2_ and 0.5 M NaCl, pH 4.0, followed by solution B containing 0.1 M Tris-HCl and 0.5 M NaCl, pH 8.0. The filtered SGM was loaded on the affinity column, incubated overnight at 4°C, and then washed with a buffer (0.1 M borate, 0.5 M NaCl, and 0.05% Tween-20). The nTyr-p3 was eluted with an elution buffer (0.1 M glycine, 0.15 M NaCl, pH 2.6), and neutralized with 1/10 volume of 1 M Tris-HCl, pH 8.0.

### Protease Activity Assay

Specific trypsin activity was analyzed using p-toluene arginine-methyl ester (TAME) (Sigma-Aldrich, St. Louis, Mo., USA); the absorption was measured at 405 nm (33). The following protease inhibitors were used for the determination of specific protease activity: phenyl methyl sulfonyl (PMSF) for serine protease, dithiothreitol (DTT) for cysteine protease, and soybean trypsin inhibitor (SBTI) and tosyllysine chloromethyl ketone (TLCK) for trypsin. All protease inhibitors were purchased from Sigma-Aldrich Chemicals (St. Louis, Mo., USA). The 1.74g of PMSF was dissolved in Dimethyl sulfoxide (DMSO) to a final volume of 100ml as 1M PMSF stock solution. Test samples were pretreated with 10 mM of each inhibitor for 60 min at 25°C before the addition of the TAME substrate (11).

### Human Airway Epithelial Cell Culture

The epithelial cells derived from a human airway alveolar basal cell line, A549, were purchased from Taiwan Bioresource Collection and Research Center. The cell line A549 is widely used as an epithelial cellular model for evaluating the allergen challenge *in vitro* assays (34). A549 cells were cultured in growth media containing RPMI 1640 supplemented with 10% fetal bovine serum, 2 mM L-glutamine, 100 U/ml of penicillin, and 100 μg/ml of streptomycin. Cells were plated at a density of 2 x 10^5^ and maintained in 9 cm^2^ culture dishes at 37°C in a humidified atmosphere with 5% CO_2_. The confluence of cells at a density of 1 x 10^6^ (cells grown to confluence about 80%) was prepared for the treatment experiment. The epithelial cells were detached from the dishes by non-enzyme cell dissociation solution (Invitrogen, Thermo Fisher Scientific Inc. Waltham, MA., USA) to exclude the proteolytic activation of PARs.

### Allergen Stimulation

Before the allergen treatments, A549 cells were plated in 6-well (35 mm) culture plates in growth media for 24 h to allow attachment and starvation in serum-free media. All subsequent experiments of allergen stimulation or inhibitor treatment were performed in the serum-free condition. For the dose-concentration assessment, cells were treated with nTyr-p3 (1 to 5 μg/ml) for 2 h. Untreated cells served as a negative control. For the allergen-stimulation (nTyr-p3) assessment, the levels of PAR-1, PAR-2, PAR-3, and PAR-4 mRNA were evaluated. The synthetic peptide of 2f-LIGRLO-NH_2_ (1µM) was used as a PAR-2 agonist (35, 36). At the end of each treatment, epithelial cells were detached, and viability and cell number were determined by the trypan blue exclusion method. Cells were pretreated with various protease inhibitors (5mM of PMSF, DTT, and SBTI) for 60 min at 25°C before allergen treatment. Cells were also treated with inhibitor alone of PMSF, DTT, and SBTI and solvent alone of DMSO as controls.

### Extraction of RNA and Quantitative Reverse Transcription Polymerase Chain Reaction (RT-qPCR) Analysis

Total RNA from treated A549 cells was extracted using RNAzol reagent according to the manufacturer’s instructions (Invitrogen, Thermo Fisher Scientific Inc. Waltham, MA., USA). The concentration and purity of RNA were analyzed and determined with absorbance at a wavelength of OD260 and 280nm by NanoDrop ND-1000 spectrometer (Thermo Fisher Scientific Inc. Waltham, MA., USA). First-strand cDNA was synthesized from 1 µg of total RNA by reverse transcription using a SuperScript III first-strand cDNA synthesis kit according to the manufacturer’s instructions (Thermo Fisher Scientific Inc. Waltham, MA., USA). The receptor variations of PAR1 to PAR4 were evaluated by RT-qPCR analysis. Messenger RNA (mRNA) levels were quantified with cDNA by TaqMan polymerase chain reaction analysis using GeneAmp 7000 Sequence Detection System (Applied Biosystems, Uberlingen, Germany). PAR-specific primers of humans used in this study were purchased from Applied Biosystems as previously reported (37, 38). β-actin was used as the control gene for normalizing the RNA content of each sample. The detecting probe has a fluorescent reporter dye (FAM) linked at 5’end and downstream a quencher dye (TAMRA) linked at 3’end. The gene expressions of IL-6 and IL-8 were performed by semi-quantitative RT-PCR. PCR amplification was performed using a Takara PCR amplification kit (Takara, Beijing, China) in a total volume of 25 µl. Primers used for PCR analyses were designed by Primer-BLAST (National Center for Biotechnology, Rockville Pike, MD, USA) and synthesized by Clontech Laboratories (Takara, Beijing, China). A suitable temperature cycle schedule for each PCR reaction was determined using an MJ Mini™ Personal Thermal Cycler (Bio-Rad, Foster, CA, USA). The primers of IL-6 and IL-8 were described in our previous study (39). Human *ACTB* (β-actin) was used as an internal control to normalize the levels of gene expression. PCR products were separated on 1.5% agarose gel, stained with ethidium bromide, and photographed with UV light. The intensities of bands were quantified using Gel-Pro Analyzer (Media Cybernetics, Silver Spring, MD, USA).

### Enzyme-Linked Immunosorbent Assay (ELISA)

A549 epithelial cells were treated with *T. putrescentiae* crude extract (10 µg/ml) or n Tyr-p3 (5 µg/ml) for different incubation periods (0, 3, 6, 12, 24 and 48 h). The selection of allergen concentration and time point were based on the previous study (11). After treatment, the cell culture media were collected, centrifuged (500g for 10 minutes), and analyzed for IL-6 (catalog No. D6050), IL-8 (catalog No. D8000C), IL-1β (catalog No. DLB50), and TNF-α (catalog No.DTA00D) production using commercial Quantikine ELISA kits (R&D Systems, Minneapolis, MN, USA) according to the manufacturer’s instructions. Absorbance at a wavelength of 450/620 nm was measured with a microplate Powerware-X340 ELISA reader (BioTek, Winooski, VT, USA). The linear regression of the standard curve was generated with different concentrations of standards provided by the ELISA kits and obtained by software program calculation (GraphPad Prism 7, San Diego, California, USA). The concentrations of the test sample were calculated based on the standard curve.

### Phosphorylation of ERK1/2 and p38 by Western Blot Analysis

A549 epithelial cells were plated in 6-well (35 mm) dishes at a density of 2 × 10^5^/well. At 24h later, cells were stimulated with nTyr-p3 (5 µg/ml) for 10 to 45 min. After stimulation, the cells were washed with ice-cold PBS, lysed in PRO-PRER™ protein extraction solution (iNtRON Biotechnology, Seoul, South Korea), and then harvested with a cell scraper. Cell lysates were centrifuged with 14,000 ×*g* for 10 min at 4°C, and the protein concentration was determined by the Bradford method (Bio-Rad Foster, CA., USA). The standard curve of protein concentration with linear regression was generated by bovine serum albumin used as standard. The protein concentrations of samples were obtained by program calculation (GraphPad Prism 7). Protein samples were subjected to separate on 12% of sodium dodecyl sulfate-polyacrylamide gel electrophoresis (SDS-PAGE) and transferred to polyvinylidene difluoride (PVDF) membrane. The membrane was blocked with 5% skim milk and washed three times with PBST (PBS containing 1% Tween-20). The membrane was incubated with primary antibodies at appropriate dilution with gentle agitation at 4°C overnight, followed by incubation with horseradish peroxidase-coupled secondary antibody (catalog No. AP308P)(1:3000 dilution, Sigma-Aldrich, St. Louis, Mo., USA). The primary antibodies used were as follows: rabbit anti-human phospho-ERK1/2 (1:2000 dilution, R&D Systems, Minneapolis, MN, USA) and rabbit anti-human phospho-p38 MAPK (1:1000 dilution, Cell Signaling, Beverly, MA, USA). The primary antibodies and secondary antibodies were diluted with PBS. Signals were visualized by enhanced chemiluminescence (ECL) and Amersham Hyperfilm ECL (GE Healthcare Life Science, Chicago, IL., USA). After the detection of the phosphorylation signal, the membranes were stripped with stripping buffer (catalog No. 21059)(Thermo Fisher Scientific Inc. Waltham, MA., USA) and reprobed with total anti-ERK1/2 (1:5000, R&D Systems, Minneapolis, MN, USA) and p38 MAPK (1:1000, Cell Signaling, Beverly, MA, USA) for determination of the relative intensity (34). The Western blot signals were quantified by VisionWorks LS software of UVP BioSpectrum™ 500 Imaging System (Thermo Fisher Scientific Inc. Waltham, MA., USA).

### Intracellular Calcium Measurement

Detailed procedures mainly referred to the previous study (35). In brief, the A549 epithelial cells were grown to 80% confluence. Then, cells were seeded and plated at approximately 5 × 10 ^4^ cells per well in a 96-well black wall, clear bottom. At the beginning of the experiment, the cell culture supernatant was removed and cells were incubated in dye loading buffer (Hank’s Balanced Salt Solution: HBSS with 4µM Fluo-3, 0.04% pluronic acid, 1% FBS, and 2.5mM probenecid) for 1hr at 37°C. Cells were washed with HBSS and transferred to a FLIPR Tetra instrument (Molecular Device, Sunnyvale CA, USA) for fluorescence measurements after treatment of PAR-2 agonist (2f-LIGRLO-NH_2_), PAR-2 antagonist (GB88), and allergen (nTyr-p3). Stimulants were added 10sec after reading began at various concentrations. Fluorescence was measured in real-time using excitation at 480 nm and emission at 520 nm. Calcimycin A23187 (Thermo Fisher Scientific Inc. Waltham, MA., USA) was used to measure maximum fluorescence, with individual results normalized accordingly.

### Inhibition of Proteolytic Activity, MAPK, and PAR and Their Effect in the Secretion of Inflammatory Mediators

The protease activity of nTyr-p3 was inactivated by pre-treatment with specific protease inhibitors at 5mM of each inhibitor for 60 min. The protease inhibitors included PMSF (serine protease inhibitor), DTT (cysteine protease inhibitor), SBTI, and TLCK (trypsin inhibitor). To determine the role of MAPK in the secretion of the nTyr-p3-induced inflammatory mediators, A549 epithelial cells were pretreated with MAPK/ERK inhibitor (U0126), p38 inhibitor (SB203580), ERK inhibitor (LY3214996) at a concentration of 0 to 50 μM at 37˚C for 2 h, then exposed to n Tyr-p3 (5 µg/ml) for 24 h. To test the effect of PAR-2 in the secretion of nTyr-p3-induced inflammatory mediators, cells were pretreated with PAR-2 antagonist (GB88, 10µM, Cat. No. HY-120261)(MedChemExpress, Monmouth, New Jersey, USA) at 37˚C for 1 h as previously described. The releases of inflammatory mediators including GM-CSF, VEGF, COX-2, TSLP, and IL-33 from culture supernatants were collected and measured by ELISA kits (Quantikine ELISA Kits, R&D Systems, Minneapolis, MN, USA) according to the manufacturer’s instructions. The samples were tested in a 96-well microplate, and the absorbance was read at 450 nm.

### Expression and Purification of Recombinant Allergens of *T. putrescentiae*

The major allergens of *T. putrescentiae* (Tyr-p2 and Tyr-p3) were used to determine their effect on the production of pro-inflammatory cytokine and clarify the synergy effects in the allergic subjects. Recombinant Tyr-p2 and Tyr-p3 were expressed in *E.coli* and purified as described previously (40). Briefly, an amplified DNA fragment of PCR products was cloned into a pQE30 expression vector and transformed into *Escherichia coli*-M15. Expression of the recombinant allergens was performed according to the QIA expressionist™ kit (Qiagen, Hilden, Germany) according to the manufacturer’s instructions. Recombinant allergens were purified by nickel-nitrilotriacetic acid agarose metal affinity column chromatography under native conditions.

### Patient Recruitment and Exclusion Criteria

Twenty allergic patients were recruited from the allergy clinic of the Division of Allergy, Immunology, and Rheumatology of Taichung Veterans General Hospital. All allergic patients were sensitive to storage mite-*T. putrescentiae* and had mite-specific IgE as determined by the Phadia ImmunoCAP system (Thermo Fisher Scientific Inc. Waltham, MA., USA). The diagnosis criteria of asthma were used according to guidelines of the Global Initiative for Asthma (GINA) guideline 2018. Diagnosis of allergic rhinitis was based on Allergic Rhinitis and its Impact on Asthma (ARIA) guideline 2016. All patients were advised to stop the anti-allergy medication for at least 2 weeks prior to attending this study (those who could not stop anti-allergic drugs or with acute allergic symptoms were excluded). Ten non-allergic healthy volunteers were recruited as control. The study was approved by the Research Ethics Committee of Taichung Veterans General Hospital (TCVGH IRB No. C07126). All individuals for this study provided written informed consent. The peripheral venous blood sample was collected from recruited volunteers and was immediately processed to fractionate plasma and cells for further analysis.

### Immunostaining Analysis of Cell Junctions With a 2-Photon Molecular Excitation Microscopy

The epithelial cell line-A549 was used as a cellular model for evaluating the morphology variation after the allergen challenge. The morphological variations of tight junctions and adherens junctions were assessed by immunostaining analysis with fluorescent antibody labeling in methanol-fixed cells. Cells were grown on glass coverslips coated with an ultra-thin layer of Matrigel (BD Matrigel™ BD Biosciences, Billerica, MA, USA). Cells were washed with PBS and fixed in a 1:1 mixture of methanol and acetone at -20°C. Coverslips were treated with a blocking buffer (10% normal goat serum, 2% Triton X-100, 1% Tween-20, in PBS). The intercellular tight junction, ZO-1 protein, was immunostained using mouse anti-human ZO-1 monoclonal antibody (clone 1/ZO-1, BD Biosciences, Billerica, MA, USA) and adherens junction marker, E-cadherin, using mouse anti-human E-cadherin monoclonal antibody (clone DH01, Thermo Fisher Scientific Inc. Waltham, MA., USA). The goat anti-mouse conjugated with fluorescein isothiocyanate (FITC) or rhodamine was used as a secondary antibody (Thermo Fisher Scientific Inc. Waltham, MA., USA). After staining, the coverslips were mounted in gelvatol medium with phenylenediamine and examined with 2-photon molecular excitation microscopy (Zeiss Axiovert 200M, Berlin, Germany) using an X63 plan-apochromat oil-immersion objective. Images were taken with MicroMAX digital CCD cameras.

### Frozen Section and Immunostaining of Mite Body

Stock cultures of *T. putrescentiae* storage mites were preserved at 25°C and 75% relative humidity in an isolated mite-rearing facility as described in our previous study (11). The live mites were separated from the culture medium by a writing brush, embedded using Tissue-Tek^R^ O.C.T. ™ compound, and then stored at -70°C. The serial sections of 5 μm thickness of mite body were performed in a cryostat at -20°C and the sectioned specimens were transferred on glass slides pre-coated with poly-L-lysine. The slide was washed three times with 0.1 M PBST (pH 7.2) and then blocked for 1 h with gentle agitation followed by incubation with rabbit anti-nTyr-p3 primary antibody (1:2,500 dilution). The anti-nTyr-p3 antibody was confirmed by Western blotting with *T. putrescentiae* crude extracts, nTyr-p3, and rTyr-p3 in our previous study (11). After washing with PBST three times, the slide was incubated with the goat anti-rabbit secondary antibody (1:5,000 dilution) conjugated with FITC. The mounted coverslip was viewed under a fluorescence microscope.

### Statistical Analyses

Statistical analyses were carried out using SPSS, Version 12 (SPSS, Inc., Chicago, IL). Protease inhibition experiments were analyzed using one-way ANOVA and Dunnett’s multiple comparison tests. A *p*-value of less than 0.05 was considered statistically significant. The statistical analyses of gene and protein expressions were performed using GraphPad Prism 5 (GraphPad Software, San Diego, CA, USA). Data are presented as mean ± deviation (SD). The differences between the groups were analyzed using the Mann-Whitney U test. *p*-values < 0.05 were considered statistically significant.

## Results

### Purification and Characterization of Native Tyr-p3 (nTyr p 3)

**Figure 1:**
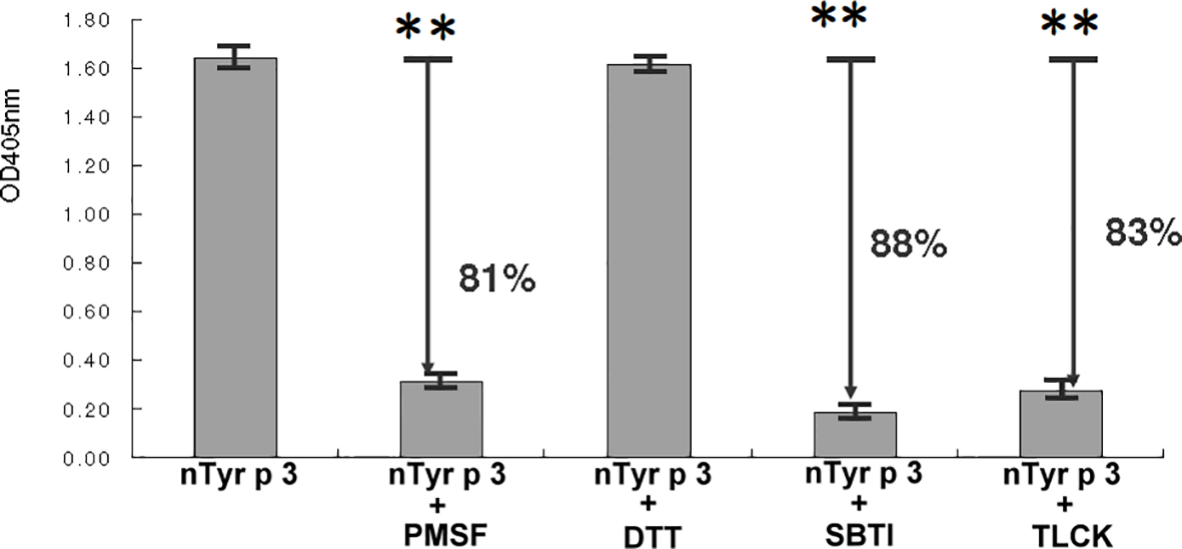
Enzymatic activity of nTyr-p3. The specific substrate of serine protease, tosyl arginine methyl ester (TAME), was used to test the serine protease activity. Four protease inhibitors were used as follows: a serine protease inhibitor (phenyl methyl sulfonyl, PMSF), a cysteine protease inhibitor (dithiothreitol, DTT), trypsin specific inhibitors (soybean trypsin inhibitor, SBTI; tosyl lysine chloromethyl, TLCK). The absorption was measured at OD405 nm. The higher absorbance value indicates a higher enzymatic activity. Arrows indicate the percentage of inhibition. Absorbance values are presented as mean ± SD. The *p*<0.01 is indicated by ** (with and without nTyr-p3).

The purified nTyr-p3 was separated on a 12% SDS-PAGE and visualized by the silver staining method (41), which showed a single band with a molecular weight of 26 kDa (**Supplementary Figure 1A**). The protein identification and peptide sequence analysis of the trypsin-digested nTyr-p3 were validated by the matrix-assisted laser desorption ionization time-of-flight mass spectrometry (MALDI-TOF MS). The identified peptide sequences were underlined (**Supplementary Figure 1B**). Several peptides were matched in the database searches, and the representative peptide sequence of VSQYLDWIELSK is shown (**Supplementary Figure 1C**). The peptide sequence of purified nTyr-p3 was characterized as identical to the accession number ABZ81991.1 in the GenBank of National Center for Biotechnology Information (NCBI) and belong to group 3 allergen of *T. putrescentiae* according to World Health Organization/International Union of Immunological Societies (WHO/IUIS) Allergen Nomenclature Sub-Committee (http://www.allergen.org/). Enzymatic activity assays showed that the nTyr-p3 was able to digest tosyl arginine methyl ester (TAME), a synthetic substrate of a serine protease. The nTyr-p3 catalyzed the substrate (TAME) to generate p-nitroaniline (*p*-NA) with enzymatic activity up to 1.6 U (μmol/min), measured at OD 405 nm after 4 h incubation at 37°C temperature (**Figure 1**). An enzyme unit (U) is defined as the enzyme activity that catalyzes the conversion of 1 micromole of substrate per minute. These results indicate that the allergen nTyr-p3 had the enzymatic activity of serine protease and that activity was significantly inhibited by serine protease inhibitors, PMSF (81% inhibition), and trypsin inhibitors, including SBTI (88% inhibition) and TLCK (83% inhibition) (**Figure 1**). However, the protease activity of nTyr-p3 was not inhibited by cysteine protease inhibitor, DTT (dithiothreitol). Additionally, nTyr-p3 could not digest N-benzoyl-tyrosine ethyl ester (BTEE) (data not shown), which is a substrate of chymotrypsin, thus indicating that nTyr-p3 did not have enzymatic activity on substrates of cysteine protease or chymotrypsin.

### Effect of the nTyr-p3 Allergen on the PAR-2 mRNA Variety in Human Alveolar Basal Epithelial Cell

**Figure 2:**
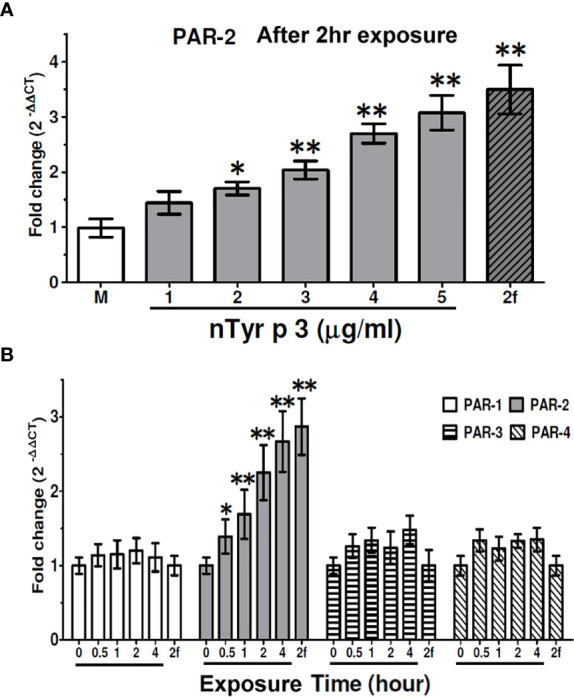
Effects of nTyr-p3 treatment on the expression of the *PAR1-4* mRNA. Total RNA was extracted from human alveolar basal epithelial cells A549 after treatment with nTyr-p3 (1 to 5 µg/ml). **(A)** Effects of nTyr-p3 treatment on the expression of the *PAR2* mRNA. *PAR-2* mRNA levels were detected by quantitative reverse transcription polymerase chain reaction (RT-qPCR) analysis. The *PAR-2* mRNA was expressed relative mRNA expression using 2^(-△△CT)^ calculation method. The *ACTB* mRNA was used as an internal control. M: Untreated cells in the medium. Lane 1 to Lane 5 with underline: treated with nTyr-p3 at 1 µg/ml to 5 µg/ml. 2f: 2f-LIGRLO-NH_2_ at 1µM (PAR-2 agonist). Data presented as the mean relative mRNA expression ± SD from three independent experiments. *p<0.05 when compared with M. ** for *p*<0.01 when compared with M. **(B)** Time-dependent effects on the levels of *PAR-1-4* mRNAs with the nTyr-p3 treatment. RT-qPCR analysis shows the mRNA levels of *PAR* 1-4 genes in A549 cells treated with nTyr-p3 (5 µg/ml) for 0 to 4 h. The result is shown as a histogram for the mRNA expression of *PARs* normalized with *ACTB* and presented as mean ± SD from three independent experiments. The exposure time is labeled with an underline. 2f: 2f-LIGRLO-NH_2_ at 1µM (PAR-2 agonist). *p<0.05 when compared with starting time. ** for *p*<0.01 when compared with starting time.

To test the effect of nTyr-p3 on the *PAR-2* mRNA expression, the human alveolar basal epithelial cells A549 were treated with different concentrations of nTyr-p3 (1 to 5 µg/ml) for 4 h, and quantitative RT-PCR was performed for mRNA variety analysis. The activating peptide (2f-LIGRLO-NH_2_) used as a PAR-2 agonist referred to other studies (35). The synthetic peptide 2f-LIGRLO-NH_2_ was used as a positive control of PAR-2 activation. As shown in **Figure 2A**, the *PAR-2* mRNA was constitutively expressed in human epithelial cells. After nTyr-p3 treatment, the levels of *PAR-2* mRNA were slightly increased by the enhanced concentration of nTyr-p3 (**Figure 2A**). After treatment with 5 µg/ml of nTyr-p3, there was a three-fold change of *PAR-2/ACTB* when compared with the medium (**Figure 2A**). Treatment of nTyr-p3 at a dose of higher than 10 μg/ml caused the detachment of the epithelial cells, which were observed as floating in the medium, thus affecting epithelial cell growth (data not shown). After treatment of PAR-2 agonist 2f-LIGRLO-NH_2_ peptide, there was over a three-fold change of *PAR-2/ACTB* when compared with medium (**Figure 2A**).

Next, we examined the effect of the nTyr-p3 (5 µg/ml) on the levels of *PAR* mRNA expression in A549 cells for different periods. The *ACTB* mRNA level was used as an internal control to normalize the *PAR* mRNA level. We observed that the *PAR-2* mRNA levels were significantly increased in the presence of nTyr-p3 for 0.5 h and remained high up to 4 h (**Figure 2B**), though the levels of *PAR-1, PAR-3*, and *PAR-4* mRNA did not obviously change. Based on the medium, the mRNA expression of *PAR-2* increased by two-folds in cells when treated with nTyr-p3 (5 µg/ml) for 2 h, by 2.5-fold change for 4 h (**Figure 2B**). Taken together, these results indicate that the allergen with nTyr-p3 activity stimulates the *PAR-2* mRNA induction, and its major receptor for functional activity of allergen nTyr-p3 is passed through PAR-2.

### Effects of Protease Inhibitors and PAR-2 Antagonist-GB88 on the nTyr-p3-Induced PAR-2 mRNA expression

**Figure 3:**
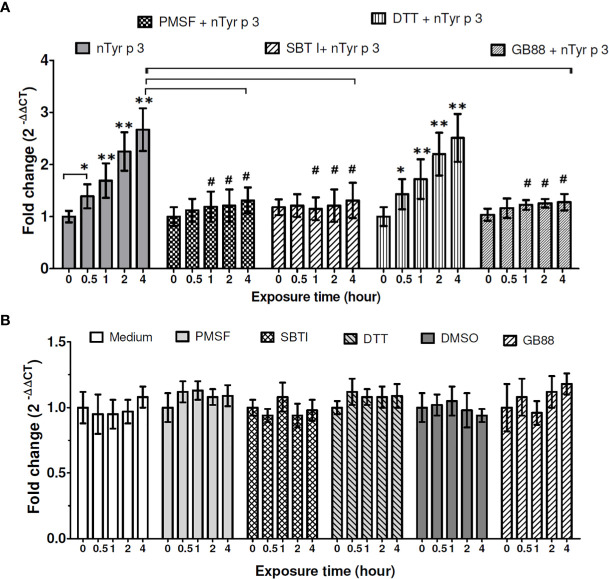
Effects of the protease inhibitors and GB88 on the nTyr-p3-induced *PAR-2* mRNA expression. The mRNA levels of *PAR-2* were performed with RT-qPCR analysis in untreated or treated A549 cells with nTyr-p3 (5 µg/ml treated) for 0 to 4 h *ACTB* was used as an internal control gene. M: untreated A549 cells in medium. PMSF, phenyl methyl sulfonyl (5mM); DTT, dithiothreitol (5mM); SBTI, soybean trypsin inhibitor (5mM). GB88: PAR-2 antagonist (10µM). DMSO: PMSF diluent (0.5% *v*/*v).* nTyr-p3 was pretreated with protease inhibitors or GB88 for 60 min at 25˚C before the addition to culture media of A549 cells. **(A)** The *PAR-2* mRNA was expressed relative mRNA expression using 2^(-△△CT)^ calculation method. Result is shown as a histogram for the mRNA expression of *PAR-2* normalized with *ACTB* and presented as mean ± SD from three independent experiments. ** for *p*<0.01 and * for *p*<0.05 when compared with untreated cells in the medium (M). # for *p*<0.05 when compared with cells treated with nTyr-p3. **(B)** Cells treated alone with protease inhibitors (PMSF, SBTI, and DTT), GB88, and DMSO. Result is shown as a histogram for the mRNA expression of *PAR-2* normalized with *ACTB* and presented as mean ± SD from three independent experiments. There were no obvious differences in the PAR-2 *mRNA* expressions after these treatments when compared to the cells in the medium (M).

The *PAR-2* mRNA expression in human epithelial cells was significantly increased by nTyr-p3 stimulation (5µg/ml) after 0.5 to 4 hours of exposure (*p*<0.05) (**Figure 3A**) (repeated data in **Figure 2B** for statistical comparison between groups). To test the protease activity of nTyr-p3 in the stimulation of the *PAR-2* mRNA levels, nTyr-p3 was pretreated with protease inhibitors (PMSF- serine protease inhibitor, SBTI- trypsin-like serine protease inhibitor, and DTT- cysteine protease inhibitor), then added to culture media of A549 cells. Results showed that the *PAR-2* mRNA levels were inhibited to a baseline when nTyr-p3 was pretreated with inhibitors, the activities were significantly inhibited by PMSF and SBTI after 1 to 4 hours of exposure (*p*<0.05) (**Figure 3A**). However, the *PAR-2* gene expression was not inhibited by cysteine protease inhibitors such as DTT (**Figure 3A**). When the nTyr-p3 was pretreated with PMSF and SBTI, the *PAR-2* mRNA expression was inhibited to baseline level, suggesting the induction of *PAR-2* mRNA expression was mediated by the protease activity of nTyr-p3. To confirm the antagonism of GB88 on the *PAR-2* mRNA levels after the allergen stimulation, the GB88 was pretreated in human epithelial cells then stimulated with nTyr-p3. The PAR-2 *mRNA* expression in A549 cells was without alternation even triggered with nTyr-p3 (**Figure 3A**), it seems that the PAR-2 *mRNA* expression could be blocked by GB88. When cells were treated alone with protease inhibitors (PMSF, SBTI, and DTT), DMSO (PMSF diluent), or GB88, the PAR-2 *mRNA* expressions were no obvious difference (**Figure 3B**). It indicates that the enzymatic activity of nTyr-p3, presumably having a trypsin-like serine protease activity, plays a critical role in the mRNA induction of *PAR-2*. The PAR-2 *mRNA* expression induced by protease allergen nTyr-p3 could be moderate when pretreated with GB88. It indicates that PAR-2 antagonism indeed could be performed by GB88.

### Protease Allergen of nTyr-p3 Induces the Levels of the Pro-Inflammatory Cytokine and Chemokine Genes

**Figure 4:**
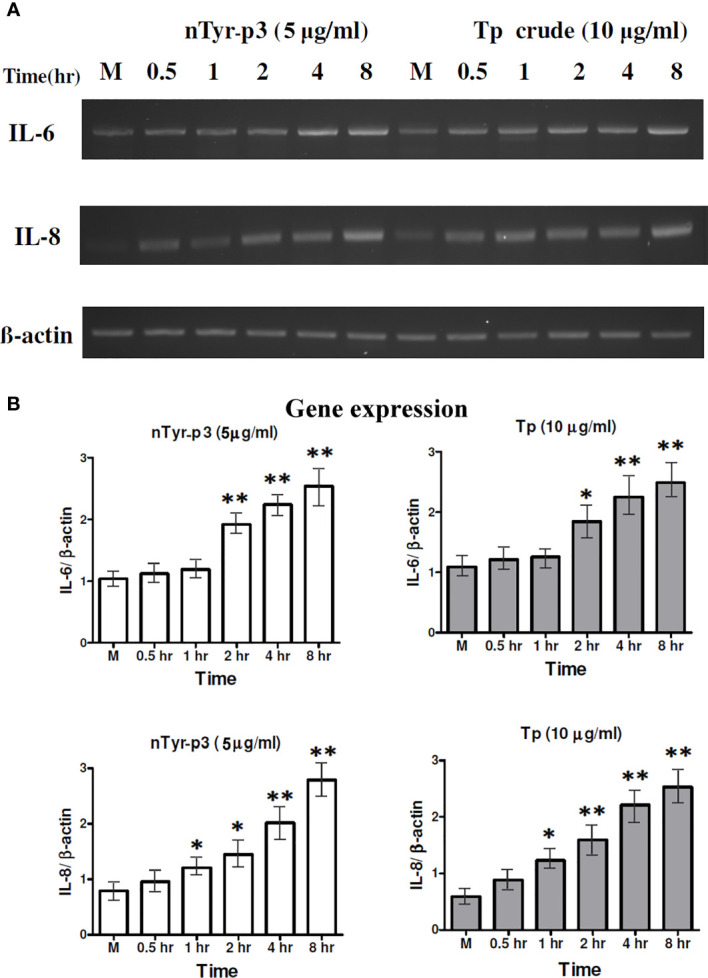
Expression of the pro-inflammatory cytokine (IL-6) and chemokine (IL-8) stimulated by nTyr-p3 and Tp crude extracts. The mRNA levels of *IL-6* and *IL-8* genes were evaluated by semi-quantitative RT-PCR after treating A549 cells with nTyr-p3 (5 µg/ml) or Tp crude extract (10 µg/ml) for 0.5 to 8 h All experiments were performed at least three times. **(A)** The representative gel image shows the expression of *IL-6* and *IL-8* genes on a 1.5% agarose gel. **(B)** The histogram shows the normalized expression levels of *IL-6*/*ACTB* as mean ± SD. ** for *p*<0.01 and * for *p*<0.05 when compared with untreated cells in the medium (M).

The effects on the productions of pro-inflammatory cytokine (IL-6) and chemokine (IL-8) of mite protease allergen were evaluated after treatment with the *T. putrescentiae* crude extracts (10 μg/ml) and nTyr-p3 (5 μg/ml) for 0.5 to 8 h in human epithelial cells. The *IL*-6 mRNA levels were significantly increased by Tp crude extracts and purified nTyr-p3 allergen at 2 h and continued high up to 8 h when compared to internal control (*p*<0.01 with nTyr-p3 and *p*<0.05 with Tp extracts) (**Figures 4A, B**). In addition, the *IL*-8 mRNA levels were also enhanced in those cells treated with nTyr-p3 (5 μg/ml) or Tp extracts (10 μg/ml) for 1 h (*p*<0.05) (**Figures 4A, B**). A two-fold increase of *IL*-8 mRNA was observed in A549 cells treated with nTyr-p3 or Tp crude extracts for 4 h (*p*<0.01) when compared with the *ACTB* mRNA levels. Thus, the protease allergens from Tp crude extracts including purified nTyr-p3 stimulated the gene expression levels of *IL-6* and *IL-8* in human airway epithelial cells.

### nTyr-p3 Triggers the Release of Pro-Inflammatory Cytokines (IL-6, TNF-α and IL-1β) and Chemokine (IL-8)

**Figure 5:**
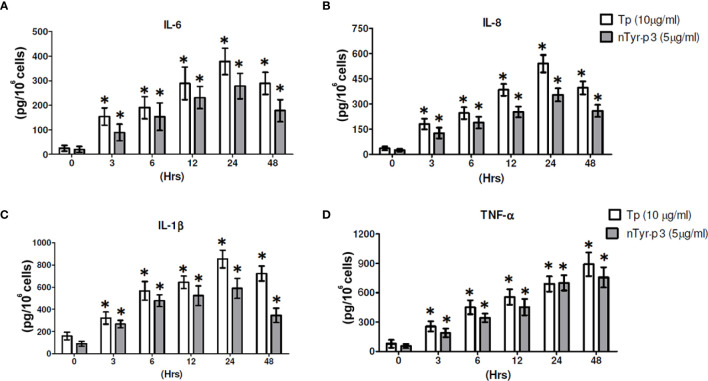
The nTyr-p3 and Tp crude extracts induce the secretion of pro-inflammatory cytokines and chemokines. The protein levels of pro-inflammatory cytokines (IL-6, TNF-α, and IL-1β) and chemokine (IL-8) were evaluated in the epithelial cells A549 after treatment with nTyr-p3 (5 µg/ml) or Tp crude extract (10 µg/ml) for 0 to 48 h The media after treatment at the indicated periods were collected for ELISA. **(A)** for IL-6, **(B)** for IL-8, **(C)** for IL-1β, and **(D)** for TNF-α levels are shown. * for *p*<0.05 when compared with untreated cells in the medium.

The pro-inflammatory cytokine production such as IL-6 or TNF-α induced by allergens is related to allergic inflammation. The chemokine of IL-8 is produced by macrophages and epithelial cells, an important mediator of the immune reaction in the innate immune system response. The production of pro-inflammatory cytokine IL-1β is involved in NLRP3 inflammasome activation and correlated with allergic airway inflammation. To understand the potential mechanism in allergy pathogenesis by nTyr-p3, we investigated the secretion of these inflammatory markers triggered by nTyr-p3 in human epithelial cells. The optimal concentrations of *T. putrescentiae* crude extract (10 µg/ml) and nTyr-p3 (5 µg/ml) for trigger assessment of pro-inflammatory cytokine releases were mainly based on previous studies (11, 42). The experiment was performed together with *T. putrescentiae* crude extract and nTyr-p3 not to compare the release amount of each other, but to examine whether both can induce inflammatory substances. Our results indicate that nTyr-p3 provoked progressive secretions on IL-6, IL-8 IL-1β, and TNF-α from 3 to 24 h (**Figures 5A** **–D**). Both nTyr-p3 and Tp crude extracts were able to increase the levels of IL-6, TNF-α, IL-8, and IL-1β in a time-dependent manner, and a significant difference occurred at 3 h and continued till 24 h (*p*<0.05; **Figures 5A–D**). Further, the TNF-α production continued to increase until 48 h with the Tp crude extract (**Figure 5D**). It indicates the protease allergens from *T. putrescentiae* crude extract or purified nTyr-p3 stimulates the productions of pro-inflammatory cytokines (IL-6, TNF-α, and IL-1β) and chemokine (IL-8).

### Effects of nTyr-p3 on the Phosphorylation of Extracellular Signal-Regulated Kinase (ERK) 1/2 and p38 Mitogen-Activated Protein (MAP) Kinase

**Figure 6:**
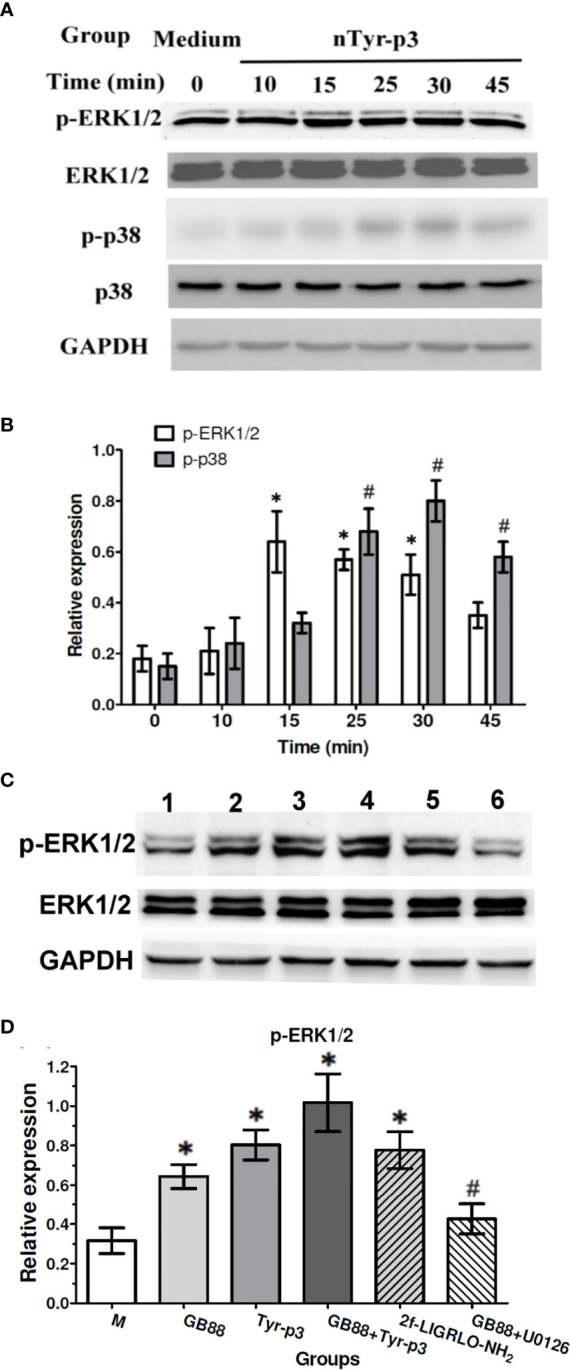
Effects of ERK1/2 and p38 MAP kinase phosphorylation after nTyr-p3 stimulation and GB88 treatment. Epithelial cells A549 were cultured in the serum-free medium for 24h before nTyr-p3 stimulation. The A549 cells were stimulated with nTyr-p3 (5 µg/ml) for 10 to 45 minutes. Protein samples from the cell lysates were separated on 12% of SDS-PAGE and transferred to PVDF membranes. Equal amounts of proteins were used for Western blotting using GAPDH as a loading control for internal control of protein levels. All experiments were performed at least three times. **(A)** The representative data of phosphorylated-ERK 1/2 and p38 at different times, as indicated in the panel. **(B)** The relative levels of p-ERK1/2 and p-p38 are presented as mean ± SD with histogram. * for *p*<0.05 when compared with the total ERK1/2, and # for *p*<0.05 when compared with the total p38. **(C)** The phosphorylation changes were observed after 30 min treatment. A549 cells treated with various groups separately, including GB88 (10 µM), nTyr-p3 (5 µg/ml), GB88 and nTyr-p3, 2f-LIGRLO-NH_2_ (1µM), and GB88 + U0126 (10 µM). The cells were pretreated with GB88, then stimulating with nTyr-p3 were obtained for Western blotting. 2f: 2f-LIGRLO-NH_2_: PAR-2 agonist. U0126: MAPK/ERK inhibitor. The representative data of phosphorylated-ERK 1/2 was shown. **(D)** Result was shown as a histogram and presented as mean ± SD from three independent experiments. *p<0.05 when compared with untreated cells in the medium. #*p*<0.05 when compared with GB88 alone.

MAP kinases (MAPKs) are involved in controlling cellular responses through diverse stimuli, including proinflammatory cytokines. The MAPK/ERK pathway communicates a signal from the cell surface receptor to the DNA in the nucleus of the cell. It is reported that German cockroach extracts with protease activity trigger the IL-8 release from airway epithelial cells by stimulating PAR-2 and ERK (34). To determine whether the MAP kinase signaling pathway is triggered by the allergen exposure which with protease activity, we evaluated the total and phosphorylated ERK1/2 and p38 MAP kinase proteins after activation of PAR-2 by nTyr-p3 in human A549 cells. The expression levels of total ERK1/2 protein showed no significant alterations from 0 to 45 min, whereas phosphorylated ERK1/2 protein was enhanced by nTyr-p3 treatment, which peaked at 15 min and returned back to the basal level at 45 min (**Figure 6A**). Also, phosphorylation of p38 MAPK significantly increased at 25 min, peaked at 30 min with nearly a 4-fold increase, and then decreased at 45 min when compared to the untreated control (**Figures 6A, B**). When cells were treated with GB88 alone after 30 min, the phosphorylated ERK1/2 protein was slightly increased when compared to the medium (**Figure 6C**). GB88 increased ERK1/2 phosphorylation in human epithelium cells. The challenge of allergen nTyr-p3 also significantly enhanced the ERK1/2 phosphorylation. When cells were pretreated with GB88 then added nTyr-p3, the phosphorylated ERK1/2 was not inhibited by GB88, but with a magnified effect on the ERK1/2 phosphorylation. The PAR-2 agonist 2f-LIGRLO-NH_2_ also promoted the ERK1/2 phosphorylation. The GB88-induced ERK1/2 phosphorylation could be significantly inhibited by U0126 (MAPK/ERK inhibitor)(*p*<0.05) (**Figures 6C, D**).

### Effect of GB88 on the PAR-2-Biased Ca^2+^ Release Induced by nTyr-p3 Triggers

**Figure 7:**
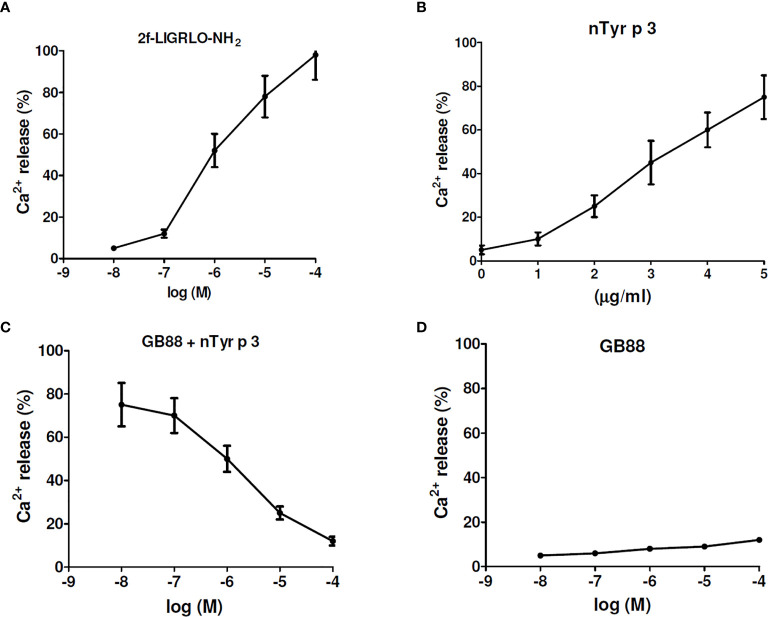
The effect of GB88 on intracellular calcium mobilization induced by allergen nTyr-p3. The intracellular calcium mobilization was evaluated in human epithelium A549 cells by a fluorescence-based binding assay. **(A)** The 2f-LIGRLO-NH_2_ (PAR-2 agonist) induced intracellular calcium release in a concentration-dependent manner (at 10^-8^M to 10^-4^M). **(B)** The allergen nTyr-p3 triggered intracellular calcium release at concentrations of 1µg/ml to 5µg/ml. **(C)** The Ca^2+^ release triggered by nTyr-p3 (5µg/ml) could be down-regulated by GB88 in a concentration-dependent manner. **(D)** The Ca^2+^ release was treated with GB88 at concentrations of 10^-8^M to 10^-4^M.

The effect of GB88 on intracellular calcium mobilization induced by allergen nTyr-p3 in human epithelium cells was evaluated by a fluorescence-based binding assay. The PAR-2-activating peptide, 2f-LIGRLO-NH_2_, could activate intracellular Ca^2+^ release at concentrations of 10^-8^ to 10^-4^ M, which the higher concentration triggers the higher amount of Ca^2+^ release (**Figure 7A**). The allergen nTyr-p3 at concentrations of 1µg/ml to 5µg/ml prompted intracellular calcium release leading to an increase of the Ca^2+^ released (**Figure 7B**). When the nTyr-p3 concentration exceeds 10µg/ml, the state of cells is obviously affected so that epithelium cells cannot be attached to the bottom smoothly and floated up. When cells were pretreated with GB88 then stimulating with nTyr-p3 at 5µg/ml, the nTyr-p3-triggered Ca^2+^ release could be down-regulated. Results indicated that the PAR-2 antagonist- GB88 is able to block PAR-2-mediated calcium signaling which inhibits the nTyr-p3-induced Ca^2+^ release (**Figure 7C**). In terms of Ca^2+^ release, the GB88 just behaved as a PAR-2 specific antagonist with no agonist activity. GB88 only slightly induced Ca^2+^ release at a high concentration of 10^-4^M in human epithelium cells (**Figure 7D**).

### Specific Pharmacologic Inhibitors and Gb88 Abolish the ntyr-p3-Induced Activation of Chemokine (il-8) and Pro-Inflammatory Cytokine (IL-1β)

**Figure 8:**
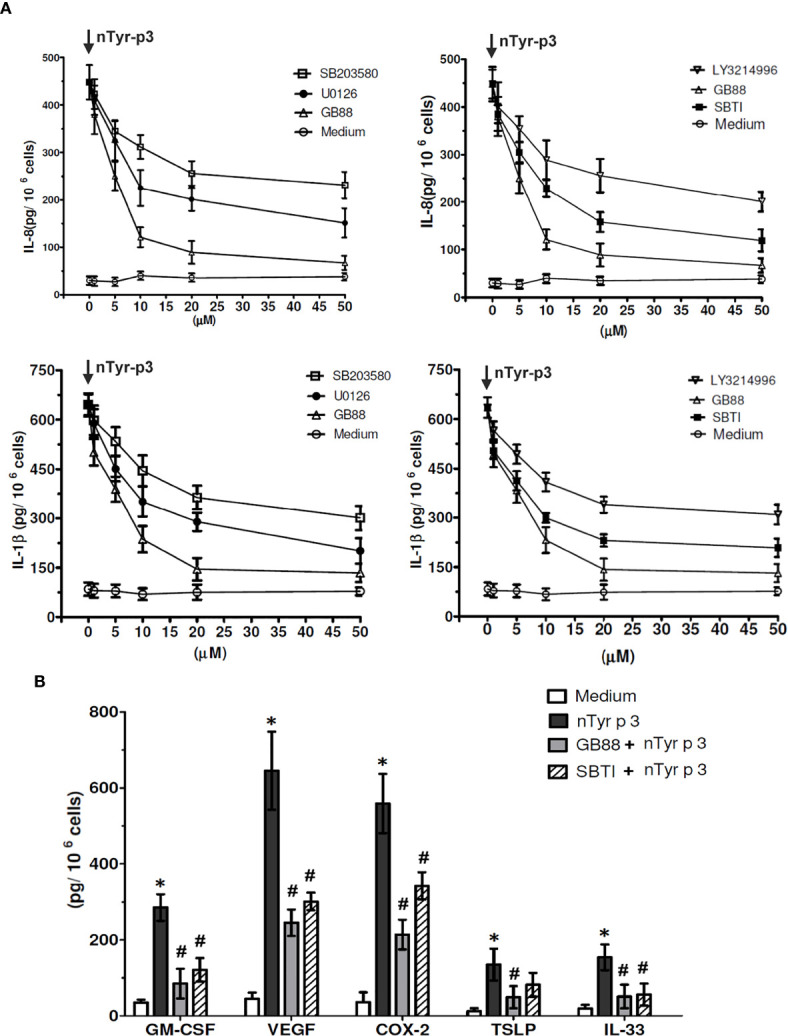
The inhibitory effects of GB88 or pharmacologic inhibitors on the nTyr-p3-activated synthesis of inflammatory mediators. **(A)** Specific pharmacologic inhibitors including SB203580 (p38 inhibitor), LY3214996 (ERK inhibitor), U0126 (MAPK/ERK inhibitor), SBTI (Soybean trypsin inhibitor), and GB88 (PAR-2 antagonist) were tested for their effects on the synthesis of IL-8 and IL-1β in A549 cells after nTyr-p3 challenge. The symbol of ↓ meant treatment with nTyr-p3. The concentrations of inhibitors were in the range of 1 to 50 µM. The levels of IL-8 and IL-1β were analyzed with 10^6^ cells, and the data is presented as mean ± SD. **(B)** Effect of GB88 or SBTI on the levels of GM-CSF, VEGF, COX-2, TSLP, and IL-33 in A549 cells. Cells were cultured with PAR-2 antagonist (GB88) and protease inhibitor (SBTI) for 4 h before the addition of the nTyr-p3 (5 µg/ml). GM-CSF, granulocyte-macrophage-colony-stimulating factor; VEGF, vascular endothelial growth factor; COX-2, cyclooxygenase-2; TSLP, thymic stromal lymphopoietin. * for *p*<0.05 when compared to the untreated cells in the medium. # for *p*<0.05 when compared with the nTyr-p3 treated cells and compared between with or without inhibitors.

Specific pharmacologic inhibitors, including SB203580 (p38 inhibitor), LY3214996 (ERK inhibitor), U0126 (MAPK/ERK inhibitor), SBTI (Soybean trypsin inhibitor), and GB88 (PAR-2 antagonist) were used to confirm the role of MAPK, ERK, and p38 activation on the production of IL-8 and IL-1β in A549 cells after treatment with nTyr-p3. The cells were pretreated with various types of inhibitors then treated with nTyr-p3. The culture media from the A549 cells were collected to measure the levels of pro-inflammatory mediators using ELISA. The most effective inhibitor on chemokine IL-8 production triggered by nTyr-p3 was GB88, which caused an 85% reduction at a concentration of 50 µM (**Figure 8A**). SBTI caused 73%, MAPK/ERK inhibitor (U0106) caused 66%, LY3214996 (ERK inhibitor) caused 56%, and SB203580 (p38 inhibitor) caused 51% reduction on IL-8 production (**Figure 8A**). Similar results were obtained in the production of IL-1β. The most effective inhibitor was GB88 (76% reduction) followed by SBTI (65% reduction), the MAPK/ERK inhibitor (64%), ERK, and p38 inhibitors (52%), all of which decreased the secretion of nTyr-p3-induced IL-1β into media. Regardless of chemokine IL-8 or pro-inflammatory cytokine IL-1β, these three types of inhibitors exhibited varying degrees of inhibition in the levels of both IL-8 and IL-1β in a dose-dependent manner (1 to 50 µM) after nTyr-p3 exposure (**Figure 8A**).

### PAR-2 Antagonist (GB88) and Protease Inhibitor (SBTI) Block the nTyr-p3-Stimulated Production of Inflammatory Mediators Including GM-CSF, VEGF, COX-2, TSLP and IL-33

To characterize the inhibitory effects of PAR-2 antagonist or protease inhibitor on nTyr-p3 stimulatory activities of inflammatory mediators, we examined whether GB88 or SBTI is involved in the reduction of GM-CSF, VEGF, COX-2, TSLP, and IL-33 in airway epithelial cells (A549). The nTyr-p3 was pre-incubated with a protease inhibitor (SBTI) to inactivate its protease activity whether affects the production of inflammatory mediators. The airway epithelial cells were cultured in the presence of PAR-2 antagonist (GB88) for 4 h then exposed to nTyr-p3. The role of GB88 in antagonistic activity against PAR-2 which inflammations excited by the protease allergen nTyr-p3 had been realized. The secretions of inflammatory mediators including GM-CSF, VEGF, COX-2, TSLP, and IL-33 were significantly increased in nTyr-p3-treated cells when compared to untreated cells (**Figure 8B**). To confirm whether the stimulatory effect of mite allergen- nTyr-p3 was due to its protease activity, we treated A549 cells with nTyr-p3 preincubated with SBTI. We found that the secretion of GM-CSF, VEGF, COX-2, and IL-33 was significantly inhibited, thus clearly suggesting that the stimulation of inflammatory mediator synthesis was mediated by the nTyr-p3 protease activity (**Figure 8B**). Further GB88 treatment antagonized the stimulatory effect of nTyr-p3 and consequently decreased the levels of GM-CSF, VEGF, COX-2, TSLP, and IL-33 (**Figure 8B**). Thus, the inhibitory effects, observed with the treatment of GB88 or SBTI, suggest that the stimulation of inflammatory mediators by protease allergen- Tyr-p3 is mediated by its proteolytic activity through the PAR-2 mRNA expression.

### Accumulative Effect of nTyr-p3 and rTyr-p2 on the Expression Levels of IL-6/IL-8/IL-1β and IL-33 Proteins

**Figure 9:**
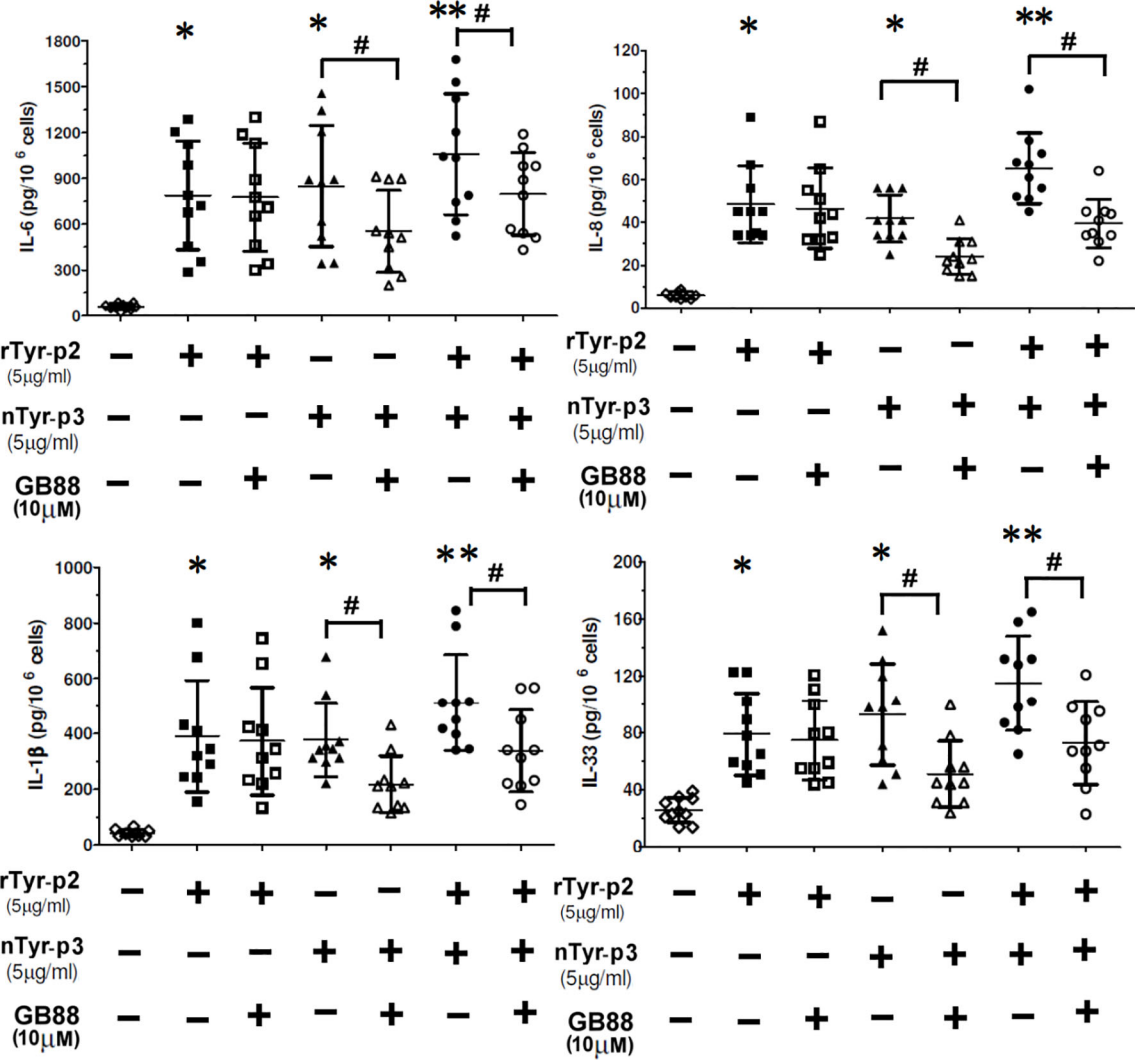
Accumulative effect of nTyr-p3 together with rTyr-p2 on the release of inflammatory mediators. Peripheral blood mononuclear cells (PBMCs) derived from ten allergic subjects sensitive to *T. putrescentiae* were pretreated with GB88 (10µM) for one hour then cultured with rTyr-p2 (5 μg/ml) or nTyr-p3 (5 μg/ml) for 24 h. Culture medium was collected and measured the levels of secreted cytokine or chemokine by ELISA. The pro-inflammatory cytokine (IL-6 and IL-1β), chemokine (IL-8), and T helper-2 associated cytokine (IL-33) expression after the allergen challenges were evaluated. Data are presented as a dot plot with the mean and SD as a horizontal line and interval. * for *p*<0.05 when compared with without allergen treatment; ** for *p*<0.01 when compared to without allergen treatment. # for *p*<0.05 when compared between with or without GB88.

Allergic diseases are associated with inflammations elicited by the production of allergen-induced pro-inflammatory cytokines. The effects of allergens on the secretion of proinflammatory cytokine or chemokine from peripheral blood mononuclear cells (PBMCs) were determined after treatment with *T. putrescentiae* allergenic components. The correlations between *T. putrescentiae* major allergens (Tyr-p2 and Tyr-p3) and allergic inflammation evaluating by PBMCs derived from ten allergic patients were cultured in the presence of rTyr-p2 (5 μg/ml) or nTyr-p3 (5 μg/ml) alone or together for 24 h. Culture media were collected to measure the levels of cytokine or chemokine by ELISA. The results showed that treatment of either rTyr-p2 or nTyr-p3 moderately increased the levels of pro-inflammatory cytokines (IL-6 and IL-1β), chemokine (IL-8), and T helper-2 associated cytokine (IL-33) (*p*<0.05; **Figure 9**). Interestingly, treatment of nTyr-p3 (protease activity) in conjunction with rTyr-p2 (non-protease) had an accumulative effect in the stimulation of these inflammatory modulators in PBMCs (*p*<0.01; **Figure 9**). The suppressive effects of GB88 against rTyr-p2 or nTyr-p3 on the pro-inflammatory cytokines (IL-6 and IL-1β), chemokine (IL-8), and T helper-2 associated cytokine (IL-33) in the allergic patients were evaluated. When cells were pretreated with GB88 (10µM) then challenged with rTyr-p2, the expressions of IL-6, IL-1β, IL-8, and IL-33 seem not suppressed by GB88 which was induced with rTyr-p2. It suggested that the inflammations triggered by non-proteolytic allergen rTyr-p2 were not affected by GB88 treatment. It also demonstrated that the Tyr-p2-induced allergic responses were not through the receptor of PAR-2. On the contrary, the productions of cytokines (IL-6, IL-1β, and IL-33) and chemokine (IL-8) induced by nTyr-p3 were significantly suppressed by GB88 (*p*<0.05; **Figure 8**). It indicated that GB88 could reduce the allergic reactions triggered by protease allergen of nTyr-p3. It seemed Tyr-p3-induced inflammations through the receptor of PAR-2.

### Effects of GB88 in Conjunction With SBTI on the PAR-2 mRNA Expression in PBMCs of the Patients With Asthma and Allergic Rhinitis

**Figure 10:**
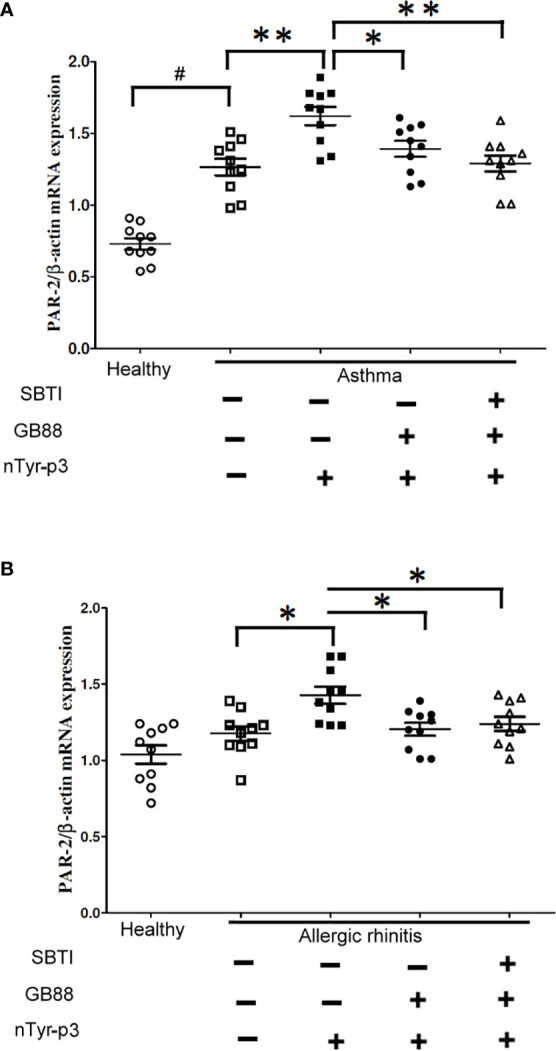
Effects of GB88 together with SBTI on the *PAR-2* mRNA levels in asthma or allergic rhinitis patients. **(A)** A comparison of the *PAR-2* mRNA levels between allergic rhinitis patients and healthy individuals was analyzed by RT-PCR. **(B)** Effects of nTyr-p3 and GB88 on the levels of *PAR-2* mRNA in PBMCs of asthma patients and healthy individuals were analyzed by RT-PCR. The *ACTB* mRNA level was used as an internal control. Normalized expression levels (*PAR-2*/*ACTB*) are presented as a dot plot with the mean and SD as a horizontal line and interval. # for *p*<0.05 when compared between healthy and allergic patients. ** for *p*<0.01 when compared with nTyr-p3 (5 μg/ml) treatment. * for *p*<0.05 when compared with GB88 treatment and ** for *p*<0.01 when compared with GB88 and SBTI treatment.

We speculated that PAR-2 might be involved in the pathogenesis of allergic airway disorders. Hence, we compared the expression of *PAR-2* in PBMCs of allergic patients with asthma, or allergic rhinitis and healthy subjects by RT-PCR. Furthermore, the stimulation with allergen nTyr-p3 and the inhibitory effects with GB88 in conjunction with SBTI on the *PAR-2* mRNA in PBMCs were also evaluated. The results showed that the *PAR-2* mRNA levels in PBMCs from allergic asthma patients were higher than in healthy subjects (*p*<0.01; **Figure 10A**). Treatment with allergen nTyr-p3 significantly increased the mRNA levels of *PAR-2* in PBMCs from patients with asthma and allergic rhinitis, when compared with that in healthy subjects. However, in the presence of PAR-2 antagonist GB88, the *PAR-2* mRNA levels were decreased in the PBMCs of allergic subjects (*p*<0.05; **Figures 10A, B**). Furthermore, treatment with GB88 together with protease inhibitor SBTI significantly reduced the expression of *PAR-2* gene particularly in patients with asthma (*p*<0.01; **Figure 10A**).

### Disruptions of Tight Junction (Zonula Occludens-1: ZO-1) and Adherens Junction (Epithelial Cadherin: E-Cadherin) by Protease Allergen nTyr-p3

**Figure 11:**
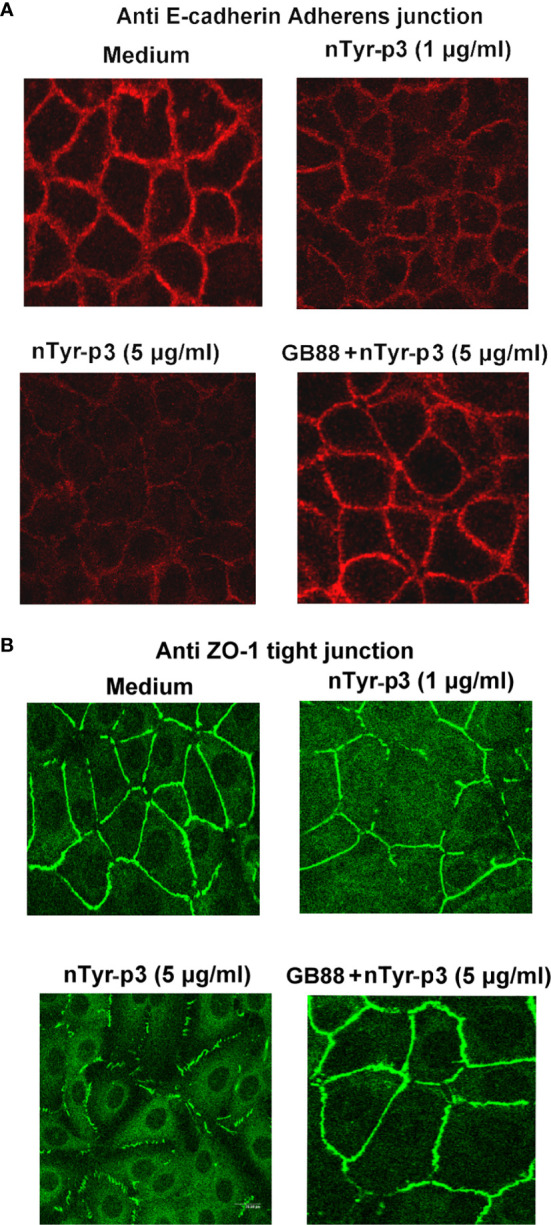
Effects of nTyr-p3 on the intercellular junctions of airway epithelium cells. Epithelial cells A549 were fixed and stained with ZO-1 and E-cadherin antibodies for indirect immunofluorescence staining. Images were taken by 2-photon molecular excitation microscopy. Cells were pretreated first with the GB88 (50 μM) and then with nTyr-p3 (5 μg/ml). **(A)** Morphology cleavages of the tight junction are shown by staining ZO-1 with green fluorescence. **(B)** Morphology cleavages of adherens junction are shown by staining E-cadherin with red fluorescence.

Both tight junctions and adherens junctions have an essential role in maintaining epithelial structure. Whether the allergen nTyr-p3 activating PAR-2 which then initiates the tight junctions and adherens junctions disruption were examined. The junction disruption could have a significant role in the pathogenesis of allergic diseases. The integrity of tight junction and adherens junction in the airway epithelium whether are disrupted by protease allergen of nTyr-p3 which possess proteolytic activity identified by previous experiment results. The morphology cleavage of intercellular junctions by immunostaining of ZO-1 and E-cadherin, markers of tight junctions and adherens junctions, respectively accomplished and analyzed by fluorescent antibody staining and 2-photon molecular excitation microscopy. Treatment of airway epithelial cells with nTyr-p3 evoked a dose-dependent decrease in ZO-1 localization at the tight cytoplasmic junction (**Figure 11A**). Treatment of nTyr-p3 at a dose of higher than 10 μg/ml caused the detachment of the epithelial cells, which were observed as floating in the medium, thus affecting epithelial cell growth (data not shown). It may be due to the proteolytic activity of Tyr-p3 with trypsin-like protease so that the epithelial cells cannot attach to the bottom of the cell culture dish. Similar results were obtained in the disruption of adherens junction by the nTyr-p3 treatment. Digital image analysis showed the intensity of E-cadherin staining apparently reduced when compared to untreated cells (**Figure 11B**). Results showed the fluorescence intensities of ZO-1 and E-cadherin were decreased, thus supporting our notion that tight junction and adherens junction were disrupted after exposure to nTyr-p3. When airway epithelial cells were pretreated with GB88 (50 μM), the morphological cleavages of tight junction (ZO-1) and adherens junction (E-cadherin) appeared to be moderately affected when compared with the nTyr-p3 treatment alone (**Figures 11A, B**). These data suggest that GB88, an antagonist of the PAR-2, probably prevented the junction disruption by counteracting the function of a proteolytic allergen in epithelial cells.

### Immunolocalization of Allergen Tyr-P3 on Mite Body

**Figure 12:**
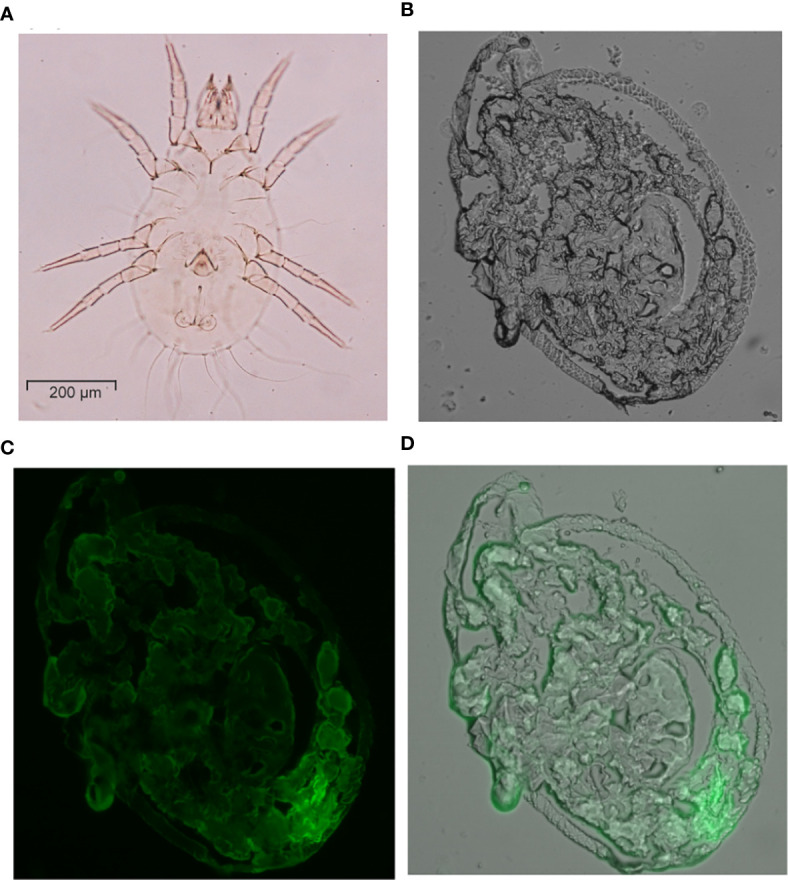
Immunolocalization of Tyr-p3 in the frozen sections of *T. putrescentiae.* **(A)** The specimens of the mite body showing the abdominal section were analyzed by an optical microscope (400x magnification). **(B)** The mite body with a longitudinal section was viewed with a visible light microscope. **(C)** Immunostaining analysis of the frozen section using a nTyr-p3 antibody. Images were taken with a fluorescence microscope. **(D)** The merged image shows the localization of Tyr-p3 (green) in the mite body (visible light), taken with visible light and green fluorescence microscopes.

The mite body sections were fixed on the glass slide and permeabilizated with Hoyer’s mounting medium for the identification of species and gender. The specimens of the mite body with abdominal section had been determined as *T. putrescentiae* male by identifying reproductive organs (**Figure 12A**). The localization of allergen-Tyr-p3 was demonstrated by the cryostat section on the mite body of *T. putrescentiae.* The longitudinal section of the mite body was observed with a bright-field microscope (**Figure 12B**). The immunolocalization was performed with the cryostat section of the mite body using an antibody against Tyr-p3. We demonstrated that the staining of allergen Tyr-p3 was observed throughout the sections in mite tissues, indicating a ubiquitous nature of this allergenic component and its biological roles in mites. Localization of Tyr-p3 was also observed throughout the intestinal digestive system, especially in the hindgut around the excretion site (**Figure 12C**). The merged image of the Tyr-p3 localization and the mite body with visible light and green fluorescence is presented in **Figure 12D**.

## Discussion

There is increasing evidence that airborne allergens are important factors in the genesis of allergic diseases including asthma and rhinitis (43). The aeroallergens derived from mites, cockroaches, and pollens may alter the integrity and function of airway epithelium, intrinsic biochemical activity, thus leading to allergic symptoms and developing airway allergy (44). The primary pattern of aeroallergen entrance into the human body is through inhalation. Interactions between airborne allergens and airway epithelial cells participate in both progress of allergic sensitization and the development of allergic airway inflammation in sensitized individuals. The inhaled allergens interact with the airway epithelial cells and cause epithelium damage, inflammatory mediator release,and airway trachea remodeling. The human pulmonary epithelial cell line, A549, generally produces similar responses to the primary human cells, indicating that A549 is a suitable cell model for studying allergen-induced airway inflammation and therapeutic evaluation. In this study, we determined whether the purified allergen nTyr-p3 affects the notable alterations on human airway epithelial cells. This is the first study, to the best of our knowledge, focusing on the allergen from storage mite *T. putrescentiae* and evaluating the effects of nTyr-p3 on epithelial cell line- A549 cells with the potential mechanism of PAR-2 in the stimulation of inflammatory mediators

The storage mite, *T. putrescentiae*, is widespread existence in urban and rural households (45), where it serves as an important source of allergen and an inducer of allergic diseases, which indicates its clinical significance. The mite allergens may come from mite bodies and feces. Because the mites spread the feces, it is considered that the proteins derived from the gut-containing feces are the most dominant source of allergens (46). Extracts of *T. putrescentiae* feces exhibit higher specific protease activities (>50-fold) than those from the mite body extracts (18). The allergenic component Tyr-p3 is detected in the feces and extract of *T. putrescentiae* by proteomic analysis, confirming further that Tyr-p3 should come from the gut-derived protein/allergen (47). It also indicates that Tyr-p3 may play an important role in the digestive physiology of the mite and has considerable relevance in allergy development (47). In the present study, the nTyr-p3 was purified from the frozen spent growth medium of mite culture, enriched with feces but devoid of the whole mites. The enzymatic properties of nTyr-p3 were characterized using a synthetic substrate of trypsin-TAME and different types of specific protease inhibitors. Our results indicated that nTyr-p3 had an enzymatic proteolytic activity similar to the serine protease. A similar observation was reported that the mite allergen Der p3 exists in the faecally enriched extracts of the house dust mite *D. pteronyssinus* and has a trypsin-like enzyme activity (33).

The human alveolar basal epithelial cell A549 was chosen to demonstrate that mite allergen nTyr-p3 was able to upregulate the *PAR-2* gene expression in a dose- and time-dependent manner. The *PAR-2* gene expression was also apparently increased after the synthetic peptide 2f-LIGRLO-NH_2_ treatment. Similar results can also be found that the peptide 2f-LIGRLO-NH_2_ represents the most potent and selective activator of PAR-2 in biological systems in the previous studies (35, 36). Similar results were also found in this research, the agonist 2f-LIGRLO-NH_2_ activates high levels of PAR-2 expression and induces calcium release in a concentration-dependent manner; however, the 2f-LIGRLO-NH_2_-induced receptor binding and Ca^2+^ release could be inhibited by GB88 (35). However, the *PAR2* activation was inhibited by trypsin-like serine protease inhibitors such as SBTI, thus suggesting that nTyr-p3 processes trypsin-like serine protease activity and it plays a critical role in the activation of PAR-2.

Among the several structural and immune cells involved in the allergen exposure, airway epithelial cells play an early role in the process through direct interactions with allergens using pattern recognition receptors such as TLRs and PARs to initiate inflammatory and immune responses in the airway. Elevation of cytosolic Ca^2+^ is an important signaling event after the mite allergen exposure, in which Ca^2+^ influx regulates a variety of functions including gene expression, cell proliferation, and differentiation in many types of cells (48). For example, the Ca^2+^ elevation regulates the production of key inflammatory cytokines including IL-6, IL-8, and TSLP. Because of the inhaled allergens produce Ca^2+^ elevations in epithelial cells, it is thought that the Ca^2+^ release-activated Ca^2+^ channels mediate their inflammatory effects by stimulating the PAR-2 receptors (48). It has been reported that Der p3 and Der p9 cleave at the N-terminal PAR-2, which results in inducing the mobilization of Ca^2+^ *via* the activation of PAR-2 in human airway epithelial A549 cells (26). Another study reported that the mite allergen Der p3 promotes the secretion of IL-8 *via* PAR-2 activation in airway epithelial cells (49). The mold allergen from Penicillium, Pen c 13, which possesses biochemical properties of alkaline serine protease, induces IL-8 expression in A549 cells *via* PAR-2 mRNA expression (50). Pen c 13 increases phosphorylation of ERK 1/2 by a Ca^2+^ -dependent pathway that is dependent on PAR-2 activation, then induces the IL-8 release in airway epithelial cells (50). The intracellular calcium release can be induced by PAR2 agonist 2f-LIGRLO-NH_2_ in a concentration manner, but it can be suppressed by PAR-2 antagonist GB88 (35).

Similar results showed that the protease allergens from American cockroach crude extract significantly induce PAR-2 mRNA upregulation, ERK1/2 phosphorylation, and IL-8 secretion in human airway epithelial cells (34). The inhibitors of serine proteases (PMSF) and ERK1/2(U0126) and blocking antibody of PAR-2 inhibit the American cockroach allergen-induced IL-8 secretion, suggesting these receptor and signaling molecules mediated the inflammatory cytokine release (34). The airborne allergens that possess prototypic proteases such as trypsin and papain may induce TSLP production dependent on PAR-2 in human airway epithelial cell line, BEAS-2B (51). The trypsin-induced TSLP expression is totally blocked by PAR-2 siRNA treatment indicates that PAR-2 is involved in protease-induced TSLP production and may facilitate the development and exacerbation of Th2 type airway inflammation (51). In addition to epithelial cells, the mite-derived serine protease activity also contributes to the pathogenesis of atopic dermatitis through cytokine release *via* PAR-2 activation in human keratinocytes (52). Similar to house dust mite allergens, the cockroach allergen extract can also activate PAR-2, through the protease activity, in mouse lung fibroblasts causing respiratory diseases (53). Although the detailed mechanisms involved in these findings are not clearly understood, the fact that PAR-2 is activated by trypsin-like serine protease from storage mite led us to believe that Tyr-p3 may have the potential to cause downstream inflammation *via* PAR-2 mRNA expression and activation.

We previously reported that the protease allergens, Der p1/Der p3, can induce the *IL-6/IL-8* mRNA levels in human bronchial epithelial cell line Beas-2B and trigger neutrophil-related allergic inflammation (54). The cockroach proteases also increase the expression of IL-8 and TNF-α in human bronchial epithelial cells *via* activation of PAR-2 and ERK, indicating that the proteolytic activity of allergen in cockroach extract has an important role in the development of airway diseases (55). Consistent with these studies, our findings showed that the production of IL-6, TNF-α, IL-8, and IL-1β were significantly increased after the exposures of Tp crude extracts and nTyr-p3. Further, For understanding the potential mechanism of protease allergen is involved in allergy pathogenesis, we investigated these inflammatory markers triggered releases by nTyr-p3 in this study. Results indicate the protease allergen from *T. putrescentiae* crude extracts or nTyr-p3 stimulates the productions of pro-inflammatory cytokines (IL-6, TNF-α, and IL-1β) and chemokine (IL-8). The stimulation with the cytokines TNF-α or IL-1α elevated the expression of PAR-2 in a dose-dependent manner in epithelial cells (56), and the PAR-2 activation promoted the release of cytokines, including IL-6 and IL-8 in epithelial cells (57). Overall, these studies suggest that the induction of the PAR-2 by protease allergen nTyr-p3 is involved in eliciting inflammatory responses and the development of allergic diseases.

The PAR-2 activation by trypsin regulates the downstream signal transduction pathways through the activation of the MAP kinase cascade (23, 58). In a previous study, we have identified the allergen nTyr-p3 to process activity as trypsin-like protease (11). The present data also indicate that the phosphorylation of ERK1/2 and p38 MAP kinase increased after nTyr-p3-mediated activation of PAR-2 in human epithelial cells. However, the IL-8 and IL-1β levels were significantly inhibited by the pharmacologic compounds, including MAPK/ERK, ERK, and p38 inhibitors. These data corroborated that Tyr-p3-mediated PAR-2 mRNA induction in the stimulation of IL-8 and IL-1β production involves the signal transduction pathways *via* the MAP kinase ERK1/2 and p38 MAPK. Further, these findings are consistent with our previous reports indicating that the mite allergen-induced secretions of IL-6 or IL-8 were inhibited by MAPK and IκB inhibitors, suggesting that MAPK, p38 MAPK, and NFκB signaling pathways may be involved in mite-induced allergic airway inflammation (39, 59). Studies have shown that the ERK and p38 MAP kinase signaling activation participates in the release of the inflammatory mediators, including IL-1β, TNF-α, and IL-8 (60, 61).

Exogenous PAR-2 activation exaggerates the inflammatory response and leads to the production of pro-inflammatory cytokines in airway epithelial cells, and has a pathogenic role in the development of airway hyperresponsiveness (AHR) and airway inflammation (62). In this study, the productions of inflammatory mediators, including GM-CSF, VEGF, COX-2, TSLP, and IL-33, were apparently induced after the protease allergen-nTyr-p3 exposure. However, in group treatment with GB88, the induction by nTyr-p3 was significantly blunted in the levels of these inflammatory mediators. Inhibition of the functions of PAR-2 by antagonist could have potential therapeutic applications for treating respiratory and inflammatory diseases (63). An important finding has been reported that GB88 inhibits the PAR2 activated Ca^2+^ release, induced by trypsin or synthetic peptide *in vitro* (28). The GB88 is also orally bio-available and effective in reducing joint inflammation of collagen-induced arthritis *in vivo* in rats (29). Supporting an important role of PAR2 in the development of airway inflammation, the *PAR2-*transgenic animals overexpressing rodents exhibit a much higher inflammatory response, whereas the *PAR2*-null mice develop considerably less inflammation in airways when compared with wild-type mice (64). The inhibitory effects of GB88 or SBTI on PAR2 activation which is induced by nTyr p3, by proteases present in mite allergens, suggest that PAR2 represents an attractive therapeutic target for allergen-induced allergic diseases.

The major allergens of *T. putrescentiae* are identified as Tyr-p2 and Tyr-p3, and both allergens may play an essential role in the pathogenesis of IgE-mediated allergic diseases in the storage mite-sensitive population (40). The protease allergenic component of house dust mite (Der p3) could activate human airway epithelium synergistically to increase the mRNA levels of *IL-6* and *IL-8* genes in the presence of non-protease allergenic component Der p2 (39). In this study, we demonstrated that there was an accumulative effect of rTyr-p2 in conjunction with nTyr-p3 on the stimulation of pro-inflammatory cytokine (IL-6 and IL-1β), chemokine (IL-8), and T helper-2 associated cytokine (IL-33) in PBMCs derived from allergic patients. Several possible reasons may explain the accumulative effect as follows. First, the mixed population of allergenic components from storage mites in the natural environment can be inhaled into the airway simultaneously. Second, the epithelial permeability barrier function may be destroyed by protease allergens and results in increased penetration of non-protease allergens and microbes, which increases the risks of sensitization to allergenic molecules and inflammatory reactions. The PAR-2 activation by mite protease allergens can also result in a delayed barrier recovery (65). Several protease allergens have been identified from dust mites, and these allergens proteolytically degrade tight junctions in human epithelial, causing the release of proinflammatory cytokines from bronchial epithelial cells, mast cells, and basophils (66). Further, these mite protease allergens may act as Th2 adjuvants and together with non-protease allergens elicit a synergistic robust Th2 response (66). The accumulative effect of mite protease allergens may promote IgE synthesis and has direct inflammatory effects on the lung epithelium, which could be a possible reason to explain why mite allergens are associated with asthma.

The PAR-2 is a G-protein coupled receptor (GPCR) associated with inflammatory responses, metabolism, cancers, and acts as a sensor for proteolytic enzymes (67). The importance of PAR-2 activation by proteases can be seen in several inflammatory diseases like asthma, arthritis, and colitis. It has been demonstrated that PAR2-activated G_q/11_ signaling pathway, Ca^2+^ release, and protein kinase c (PKC) activation are related to inflammatory responses (35). PAR-2 can also signal independently of G-proteins *via* β-arrestin1/2 and then increased ERK1/2 phosphorylation. Several studies on the PAR-2 antagonism in animal models suggest that the PAR2 can be a potential therapeutic target (29, 68). The PAR-2 antagonist GB88 is effective in treating chronic arthritis induced by collagen in rats through inhibition of macrophage infiltration, mast cell degranulation, and β-tryptase-PAR2 signaling in joint inflammation (29). GB88 is a biased antagonist of PAR-2 that selectively inhibits PAR-2/G_q/11_/Ca^2+^/PKC signaling, leading to anti-inflammatory activity *in vivo* (35). The PAR-2 mediated intracellular pathway could be downregulated by GB88, which GB88 has important anti-inflammatory properties *in vivo* after oral administration (35). The paw edema induced by PAR2 agonist in a rat animal model is inhibited by orally administered with GB88, results suggesting GB88 mechanistically dissects PAR2 signaling (35). In the CHO-hPAR2 cell, GB88 inhibited Ca^2+^ release, but activated Gi/o and increased ERK1/2 phosphorylation (35). In the human kidney tubule cells, GB88 inhibits cytokine secretion (IL-6, IL-8, GM-CSF, and TNF-α) which is mediated by PAR-2 (35). The PAR-2 antagonist, GB88, does not trigger the calcium signaling by itself and is able to block PAR-2 intermediation calcium signaling induced by tryptase (69). Similar conclusions also found in this research that GB88 does not activate intracellular calcium release by itself, but GB88 able to block nTyr-p3-triggered calcium release. GB88 increased ERK1/2 phosphorylation in human epithelium cells. The challenge of allergen nTyr-p3 also significantly enhanced the ERK1/2 phosphorylation. When cells pretreated with GB88 then added nTyr-p3, the phosphorylated ERK1/2 was not inhibited by GB88, but with a magnified effect on the ERK1/2 phosphorylation. We speculate that the GB88 is a biased antagonist of PAR-2 that selectively inhibits PAR-2/G_q/11_/Ca^2+^ signaling in the human epithelium cells.

GB88 can selectively block the PAR-2 activation, and subsequently prevents the release of a subset of the inflammatory responses (69). Asthma and allergic rhinitis are two major allergic airway diseases, the clinical relevance of PAR-2 and potential molecular mechanism in these allergic airway disorders has been confirmed. It is also known that trypsin and tryptase induce TNF-α release through an ERK-mediated mechanism (37). Consistently, we also showed that activation of PAR-2 involved in the pathogenesis of allergic airway disorders was significantly elevated in patients with asthma and allergic rhinitis, and this activation was blunted by GB88 together with SBTI in PBMCs of patients with asthma and allergic rhinitis.

The human pulmonary epithelial cell lines, HCI-H441 and A549, have been extensively used as models of the alveolar epithelium for allergen challenge or biopharmaceutical research (70). The A549 cells were isolated from a human pulmonary adenocarcinoma and exhibit a phenotype similar to type II cells due to the presence of lamellar bodies and surfactant proteins. The HCI-H441 cells formed a functional barrier with active ion transport characterized by higher resistance than A549 cells. The tight junction of ZO-1 and the adherens junction of E-cadherin in A549 maintaining epithelial structure can still be observed after the immunostaining of ZO-1 and E-cadherin (70). Similar results could be observed that the barrier integrity of tight junctions and adherens junctions are not intact of A549 cells in the medium alone. Perhaps the A549 cell line is not the most appropriate cell platform for observing junctions, but for the consistency of this research, the experiments of junctions were evaluated by A549 cells.

The major allergen of house dust mite *D. pteronyssinus* has been identified in the gut lining, gut contents, and defecated fecal pellets by immunohistochemical fluorescent probing of cryostat sections with antibody detection (71). Similar to this, our findings based on frozen section and immunostaining of mite body indicate that the major allergen of *T. putrescentiae*, Tyr-p3, was predominantly present in the intestinal digestive system of storage mite, especially in the hindgut around the excretion site. It has been shown that the most immunoreactive components of dust mite are located in the fecal pellets, mouth, and hindgut tissue by histological staining of the mite body using enzyme-labeled antibodies for immunolocalization (72). The Der f 3 allergen from mite fecal extract or mite body processes trypsin-like protease and may be involved in the digestive process of mite (15). Group 3, 6, 9 mite allergens are serine proteases and more likely to be involved in the mite digestive system. Whether in house dust mites, scabies mites, and storage mites, the serine or cysteine proteases from the digestive tract have been implicated in the skin or respiratory protein digestion. These proteases from the mite gut involved in host protein digestion offer an approach for interfering. Mite allergens possess protease activities that are thought to be secreted into the gut of the mites and excreted in fecal pellets. These allergens can transform as aerosols accumulating in the domestic environment, hence, resulting in triggers of the allergic symptoms or pathogenesis of allergy. These protease allergens play critical roles in the initiation and chronicity of allergic responses, notably through the activation of innate immune pathways (73).

At least 20 kinds of IgE-binding allergenic components of *T. putrescentiae* have been detected in allergic patient’s sera (74). Although allergen-specific CD4^+^ Th2 cells instigate the allergic responses, notably through the production of IgE directed towards mite allergens, recent studies have demonstrated that innate immune activation also plays an important role in mite-induced allergy pathogenesis. Mite allergens cannot only induce adaptive Th2-biased responses but also as strong activators of innate immune cells, including skin keratinocytes and airway epithelial cells. These mite allergens may contact or break the anatomical barrier of the mucosal membrane and then induce innate immune stimulation. The allergic responses cause through several pathways that are pathogen-associated molecular patterns (PAMPs) and damage-associated molecular patterns (DAMPs) (75). Several receptors have been clarified that are responsible for the initiation of innate immunity activation including Toll-like receptors (TLRs), C-type lectin receptors (CLRs), NOD-like receptors (NLRs), and PARs (75). The interaction or breakdown of epithelial barrier integrity facilitates allergen uptake by dendritic cells results in the upregulation of proinflammatory and Th2 cytokines/chemokines that are released not only to recruit and activate inflammatory cells but also induce Th2 differentiation. PARs are receptors activated by proteolytic cleavage of their extracellular N terminus by proteases. Storage mites and their fecal pellets include several proteolytic enzymes. The proteolytic activity of Tyr-p3 was investigated in this study which causes the PAR-2 mRNA induction, thus leading to the release of numerous inflammatory mediators. The limitation of this study, just the receptor of PAR variety was investigated after nTyr-p3 exposure. The changes of other receptors in the human airway epithelial cells after exposure to allergen nTyr-p3 are still unknown. The details of intracellular signal transduction cascades of the PAR response have been identified that containing the increased phospholipase C levels, increased intracellular Ca^2+^ levels, mitogen-activated protein kinase, and nuclear factor κB (76). The effects of the increased Ca^2+^ levels after the PAR-activation include secretion, degranulation, and smooth muscle contraction (76). PARs activation following causes edema, promotes angiogenesis and fibrosis, and enhances IgE production, leukocyte infiltration, and airway hyperresponsiveness (76). The more detailed signal transduction cascades and pathogenesis mechanisms of protease allergen nTyr-p3 need to be clarified in further studies. Activation of PARs is involved in modulating responses of the innate and adaptive immune system under physiological as well as pathological conditions (77). PARs have been found to participate in leukocyte activation and recruitment towards the site of inflammation or protease allergen exposure. PARs are also capable of regulating the production of cytokines, chemokines, and ROS by granulocytes, monocytes, or T cells in humans (77). Therefore, PARs are apparent to be an essential component of the innate and adaptive immune responses, it denotes a therapeutic potential of blocking PARs for inflammatory or allergic diseases.

In conclusion, it is suggested that the Tyr-p3 allergen from the intestinal digestive system or mite feces of mites which this protease allergen accumulates in the domestic surrounding and leads to the disruption of the airway epithelial barrier upon inhalation. The proteolytic activity of Tyr-p3 allergen causes the PAR-2 mRNA expression, facilitates the uptake of other allergens by dendritic cells in subepithelial tissues, and leads to the release of numerous inflammatory mediators (IL-6, IL-8, IL-1β, and IL-33). When cells were pretreated with GB88 then added nTyr-p3, the phosphorylated ERK1/2 did not inhibit by GB88. GB88 increased ERK1/2 phosphorylation in human epithelium cells. GB88 can block PAR-2-mediated calcium signaling which inhibits the nTyr-p3-induced Ca^2+^ release. Taken together, our findings suggest that the GB88 has therapeutic potential in exerting as an antagonist of PAR-2 expression as well as an anti-inflammatory drug, in the prevention of the allergy development triggered by mite protease allergens.

## Data Availability Statement

The raw data supporting the conclusions of this article will be made available by the authors, without undue reservation, to any qualified researcher.

## Ethics Statement 

Institutional Review Board of Taichung Veterans General Hospital (TCVGH-IRB) reviewed and approved the ethical conduct of this study (IRB No. C07126). The patients/participants provided their written informed consent to participate in this study.

## Author Contributions

Y-JW purified the allergen, performed experiments with epithelial cells, and analyzed the data. S-JY performed experiments with PBMCS and analyzed the data. C-HY performed immunostaining analysis for cell junctions using the frozen sections of the mite body. J-JT diagnosed allergic subjects, collected patients’ sera, and assisted in experimental design. E-CL designed experiments, supervised the project, wrote, and revised the manuscript. All authors contributed to the article and approved the submitted version.

## Funding

This study was supported in part by the research grants (MOST 105-2320-B-715-004 and MOST 106-2320-B-715-005-MY3) by the Ministry of Science and Technology, Taiwan, Republic of China. This project was also supported by the grants (1051B27 and 1071B15) from MacKay Medical College, and by the grant (MMH-MM-10716) from the MacKay Memorial Hospital, New Taipei City, Taiwan.

## Conflict of Interest

The authors declare that the research was conducted in the absence of any commercial or financial relationships that could be construed as a potential conflict of interest.

## Publisher’s Note

All claims expressed in this article are solely those of the authors and do not necessarily represent those of their affiliated organizations, or those of the publisher, the editors and the reviewers. Any product that may be evaluated in this article, or claim that may be made by its manufacturer, is not guaranteed or endorsed by the publisher.

## Supplementary Material

Supplementary Figure 1 **(A). (B)** MALDI-TOF Mass Spectrometry. MALDI-TOF mass spectrometry analysis of the purified nTyr-p3. The amino acid sequence of the purified nTyr-p3 is displayed in panel **(A)** with the mass spectrometry identified sequences underlined. **(C)** Tandem Mass Spectrometry (MS/MS) of purified nTyr-p3. The identified sequences were labeled on corresponding mass spectrometry peaks in panel **(B)**. The representative peptide sequence of VSQYLDWIELSK is shown.
