# Supplementary figures and images for: Antagonism of Protease Activated Receptor-2 by GB88 Reduces Inflammation Triggered by Protease Allergen Tyr-p3
Source: Front Immunol. 2021 Sep 8;12:557433. doi: 10.3389/fimmu.2021.557433 (PMC8456102; doi:10.3389/fimmu.2021.557433)

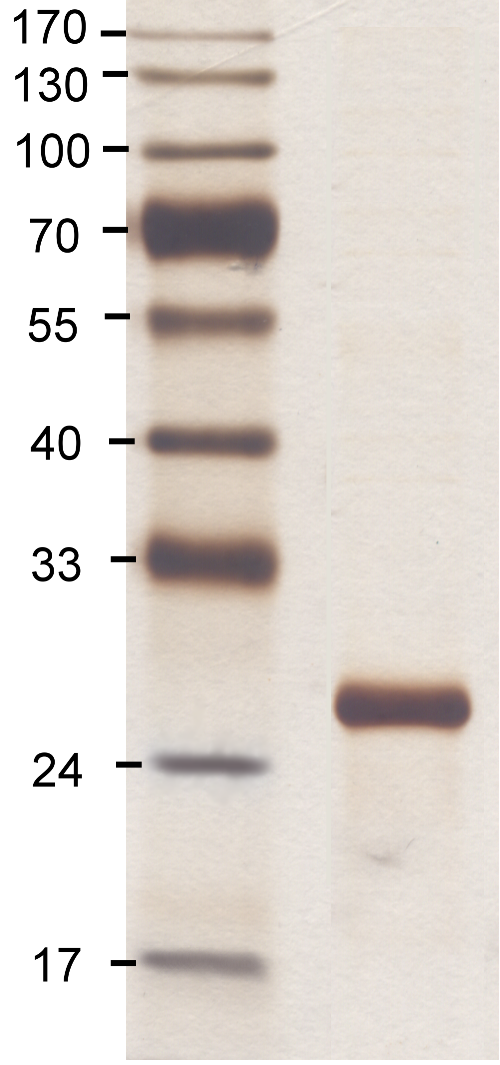

Supplement: Supplementary Figure 1 — (A). (B) MALDI-TOF Mass Spectrometry. MALDI-TOF mass spectrometry analysis of the purified nTyr-p3. The amino acid sequence of the purified nTyr-p3 is displayed in panel (A) with the mass spectrometry identified sequences underlined. (C) Tandem Mass Spectrometry (MS/MS) of purified nTyr-p3. The identified sequences were labeled on corresponding mass spectrometry peaks in panel (B). The representative peptide sequence of VSQYLDWIELSK is shown. [file Image_1.tif]

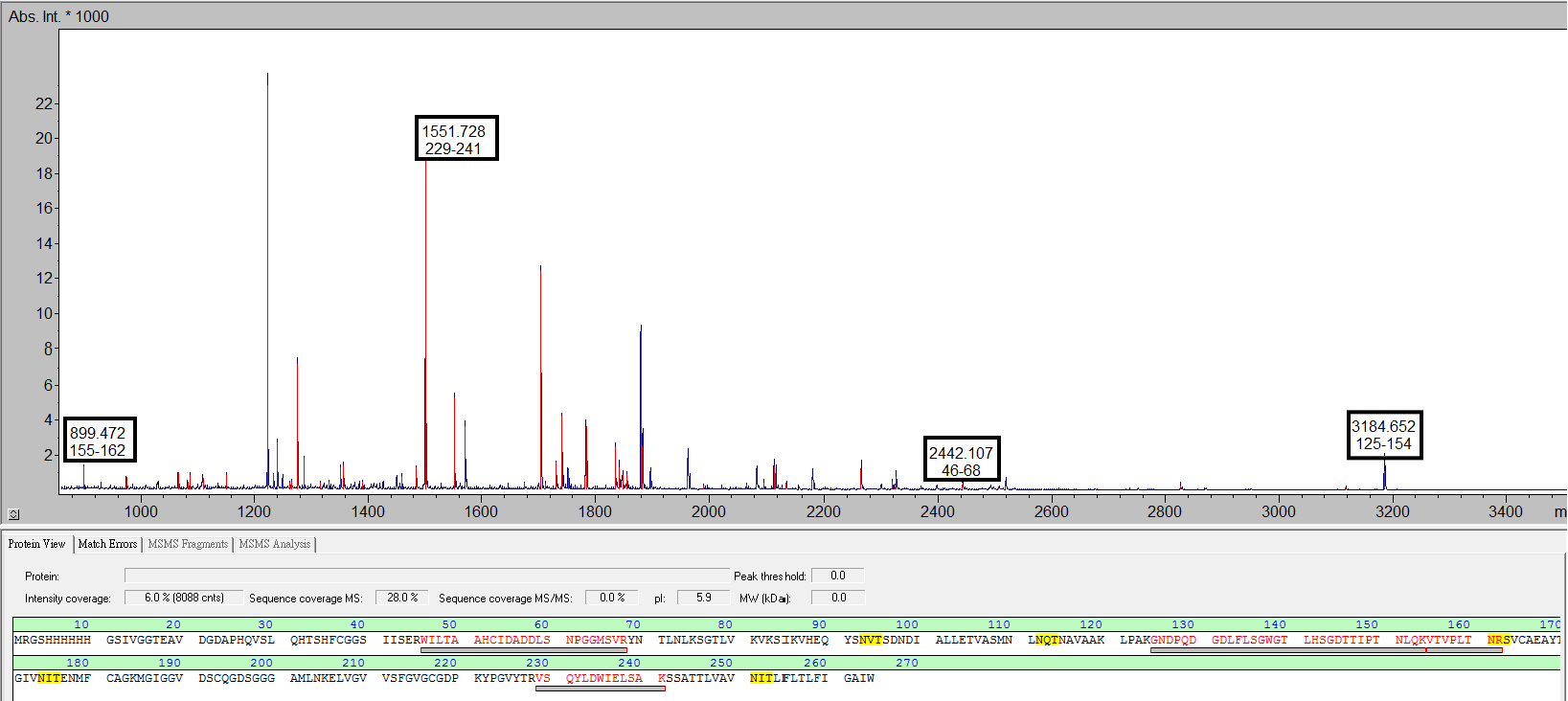

Supplement: Supplementary file 2 [file Image_2.tif]

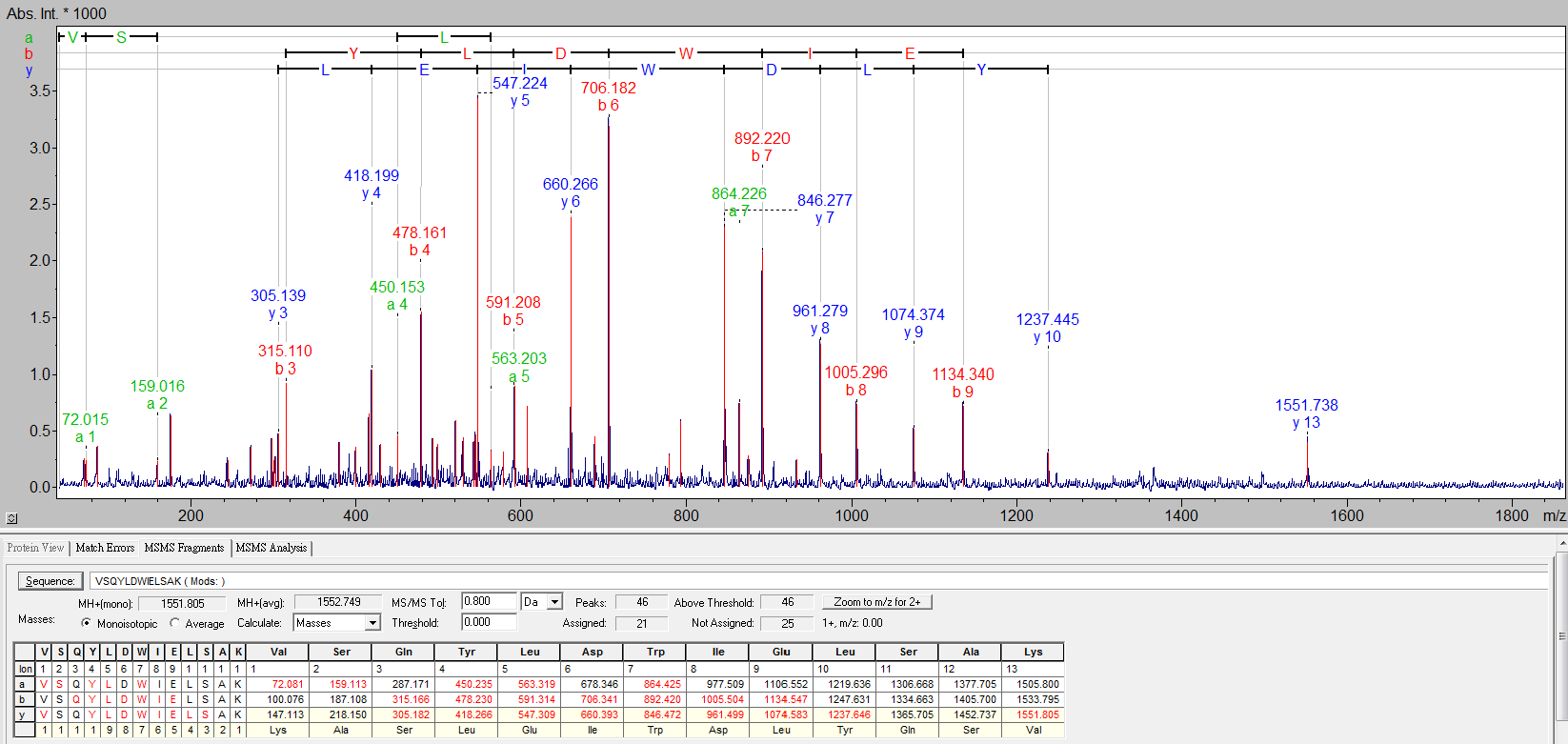

Supplement: Supplementary file 3 [file Image_3.tif]
